# Optimizing Burn Wound Healing: The Critical Role of pH and Rheological Behavior in Plant-Derived Topical Formulations

**DOI:** 10.3390/pharmaceutics17070853

**Published:** 2025-06-29

**Authors:** Oana-Janina Roșca, Georgeta-Hermina Coneac, Roxana Racoviceanu, Alexandru Nistor, Ioana-Viorica Olariu, Ana-Maria Cotan, Roxana Negrea-Ghiulai, Cristina Adriana Dehelean, Lavinia Lia Vlaia, Codruța Marinela Șoica

**Affiliations:** 1 Department of Pharmacology-Pharmacotherapy, Faculty of Pharmacy, Victor Babes University of Medicine and Pharmacy, Eftimie Murgu Square, No. 2, 300041 Timisoara, Romania; oana-janina.rosca@umft.ro (O.-J.R.); roxana.ghiulai@umft.ro (R.N.-G.); codrutasoica@umft.ro (C.M.Ș.); 2 Discipline of Clinical Practical Skills, Department I Nursing, Faculty of Medicine, Victor Babes University of Medicine and Pharmacy, Eftimie Murgu Square, No. 2, 300041 Timisoara, Romania; 3 Department of Pharmaceutical Technology, Formulation and Technology of Drug Research Center, Victor Babes University of Medicine and Pharmacy, 300041 Timisoara, Romania; coneac.georgeta@umft.ro (G.-H.C.); olariu.ioana@umft.ro (I.-V.O.); mut.anamaria@umft.ro (A.-M.C.); vlaia.lavinia@umft.ro (L.L.V.); 4 Department of Pharmaceutical Chemistry, Victor Babes University of Medicine and Pharmacy, Eftimie Murgu Square, No. 2, 300041 Timisoara, Romania; 5 Research Center for Experimental Pharmacology and Drug Design (X-Pharm Design) Victor Babes University of Medicine and Pharmacy, Eftimie Murgu Square, No. 2, 300041 Timisoara, Romania; 6 Plastic Surgery Department, University Hospital UZ Brussel, Vrije Universiteit Brussel, 1090 Brussels, Belgium; alexandru.nistor@uzbrussel.be; 7 Department of Toxicology, Drug Industry, Management and Legislation, Faculty of Pharmacy, Victor Babes University of Medicine and Pharmacy, Eftimie Murgu Square, No. 2, 300041 Timisoara, Romania; cadehelean@umft.ro; 8 Research Center for Pharmaco-Toxicological Evaluations, Faculty of Pharmacy, “Victor Babes” University of Medicine and Pharmacy Timisoara, Eftimie Murgu Square No. 2, 300041 Timisoara, Romania

**Keywords:** wound-healing properties, burn wound treatment, rheological properties, physicochemical characterization, plant extracts, hydrogel, oleogel, pH, temperature, experimental rat burn model

## Abstract

**Background:** In burn injuries, wound healing effectiveness is complex and influenced significantly by the local biochemical environment and the physicochemical properties of topical preparations. pH lesions modulation can influence protection barrier integrity, inflammatory responses, and microbial colonization. Their antioxidant, antimicrobial, and anti-inflammatory properties, of the topical formulations enriched with plant extracts have demonstrated promising results. **Objective:** The aim of the study was to develop and characterize topical oleogel and hydrogel formulations containing ethanolic and hydroalcoholic extracts of medicinal plants (*Boswellia serrata*, *Ocimum basilicum*, *Sambucus nigra*, and *Galium verum*), and to evaluate the impact of their physicochemical properties, rheological behavior, in contrast with the wound pH modulation, and healing efficacy in an experimental burn model. **Methods:** Second-degree burns were induced uniformly on Wistar rats using the validated RAPID-3D device. All formulations were applied daily for 21 days, and wound healing was assessed through several measurements specific to the wound surface, skin temperature, pH, and, last but not least, histological analyses. Formulations’ physicochemical and rheological properties, including pH, viscosity, and spreadability, were also analyzed and systematically characterized. **Results:** Oleogel formulations demonstrated superior wound healing performance compared to hydrogels. Formulations containing *Boswellia serrata* and *Ocimum basilicum* extracts significantly reduced wound size, inflammation, and melanin production by days 9 and 21 (*p* < 0.05). The beneficial outcomes correlated strongly with formulation acidity (pH < 6), high viscosity, and enhanced thixotropic behavior, indicating improved adherence and sustained bioactive compound release. Histological evaluations confirmed enhanced epithelialization and reduced inflammation. **Conclusions:** Particularly *Boswellia serrata* and *Ocimum basilicum* in oleogel formulations in ethanolic solvent effectively modulated wound pH, enhanced topical adherence, and improved burn wound healing. These findings highlight their potential clinical application and justify further clinical investigations.

## 1. Introduction

Optimal burn wound healing is a complex, multifactorial process influenced by the local biochemical environment and the physicochemical properties of topical preparations [1,2]. Healthy skin, particularly the stratum corneum, maintains an acidic pH level of 4.1–5.8, which is crucial for preserving its structural integrity and preventing microbial infections. Elevated wound pH disrupts the protective skin barrier, increasing susceptibility to infections and prolonging inflammation, consequently delaying the healing process [3,4].

Recent developments in herbal topical formulations designed to regulate wound pH have gained considerable attention in the pharmaceutical industry. These formulations aim to restore and maintain a physiologically acidic environment, thus facilitating epidermal barrier repair and mitigating inflammation [5,6]. Compared to synthetic preparations, plant-derived formulations are especially promising due to their synergistic antioxidant, antimicrobial, and anti-inflammatory properties, which collectively accelerate skin regeneration [7].

In addition to pH, monitoring wound temperature fluctuations provides valuable indirect insights into wound status, as abnormal temperature variations can signal inflammation, infection, or ischemia. Therefore, temperature measurement, alongside pH monitoring, can serve as an essential indicator in effectively evaluating wound healing progression [8].

Taking these into consideration, the selected medicinal plants—*Boswellia serrata*, *Ocimum basilicum*, *Sambucus nigra*, and *Galium verum*—were chosen for their rich profiles of bioactive secondary metabolites beneficial in wound management. *Sambucus nigra* flowers and bark contain flavonoids such as rutin and isoquercitrin, alongside phenolic acids like chlorogenic acid, recognized for their antioxidant, anti-inflammatory, antimicrobial, and skin-protective properties [9,10].

*Boswellia serrata resin* (frankincense) contains pentacyclic triterpenoids, mainly boswellic acids, known for their potent anti-inflammatory, analgesic, and antioxidant activities. These compounds support wound repair by modulating inflammation and oxidative stress [11,12].

*Galium verum* (lady’s bedstraw) aerial parts contain flavonoids and iridoid glycosides, particularly asperuloside, which are traditionally valued for their antioxidant and anti-inflammatory activities, contributing to improved skin regeneration and chronic wound management [13,14].

Similarly, *Ocimum basilicum* (basil) leaves and aerial parts are rich in phenolic acids, especially rosmarinic and caffeic acids, as well as essential oils containing linalool and eugenol. These components demonstrate pronounced antimicrobial, anti-inflammatory, antioxidant, and epithelial regeneration effects, substantially enhancing wound healing [15].

Considering these bioactive profiles, the current study aims to systematically develop and characterize topical oleogels and hydrogels incorporating ethanolic and hydroalcoholic extracts from these medicinal plants. To position our work within the existing literature, we reviewed prior studies involving plant-derived hydrogels, highlighting comparative insights into their efficacy and characteristics. Oleogel and hydrogel formulations loaded with the active components of plants extracts, optimized physicochemical and rheological characteristics could represent an effective solution to improve the personalized treatment of post-burn wounds [16,17].

Our formulations were evaluated for their physicochemical and rheological properties, focusing specifically on their ability to modulate wound pH and temperature within an established experimental burn model. To ensure reproducibility and standardization, we utilized the previously validated RAPID-3D (Rat Printed Induction Device—3D) device to create uniform second-degree burns in Wistar rats. We hypothesized that maintaining an acidic wound environment without adjusting the initial pH, combined with an optimized rheological behavior of the formulations, would significantly improve wound healing outcomes compared to standardized cream bases without incorporated extracts.

To test this hypothesis, the following section describes in detail the materials used and the methods employed in the formulation, physicochemical characterization, and evaluation of wound healing properties of the developed oleogels and hydrogels.

## 2. Materials and Methods

### 2.1. Plant Materials and Extraction

The general extraction procedure for the other plant materials used (*Boswellia serrata*—resin granules, *Ocimum basilicum* and *Galium verum*—aerial parts, and *Sambucus nigra* flowers) was previously described in detail by Rosca et al. (Pharmaceutics, 2025). Briefly, the procedure involved grinding, extraction with absolute ethanol or 70% ethanol (70:30 = ethanol: H_2_O), homogenization, sonication, filtration, rotary evaporation, and storage under recommended standard conditions [18].

#### Harvesting and Preparation of Plant Material (Sambucus Nigra Bark)

Young bark branches of Sambucus nigra were harvested during May–June, from the rural area of Lelești, located in southern Romania, from an isolated region from direct sources of industrial or urban pollution, using individual, healthy plant specimens. The harvesting was carried out by sectioning the branches located at a height of approximately 50–60 cm from the ground, selecting branches aged 1 year, with a diameter of approximately 2 cm, and an average length of 2 m.

The bark of the branches was removed by scraping, using a sterilized blade, to prevent plant material contamination. Subsequently, the obtained bark was stored in airtight “zipper” type bags and placed in suitable containers for slow drying. This procedure ensures the preservation of the bioactive properties of the plant material and the avoidance of further degradation or microbiological contamination [19].

This harvesting and preparation procedure for Sambucus nigra bark is explicitly described here for the first time.

For *Sambucus nigra* bark extraction, a novel two-step extraction method was developed and described here for the first time. Initially, dried and finely ground bark material was extracted with 900 mL chloroform, following similar extraction steps (stirring at 30 °C for 2.5 h, homogenization for 48 h, sonication at 30 °C for 1 h, refrigeration at 4 °C for 3 days, under vacuum filtration). This first step was performed in order to remove the lipophilic compounds present in the bark. The resulting vegetal material underwent a subsequent extraction with absolute ethanol (900 mL), following the same extraction steps as the other plant material used. The obtained extract was stored at room temperature in airtight containers until further use.

### 2.2. Phytochemical Profile of Extracts

The extracts were analyzed by HPLC to identify the active compounds rosmarinic acid, caftaric acid, gentisic acid, chlorogenic acid, caffeic acid, p-coumaric acid, ferulic acid and sinapic acid, hyperoside, isoquercitrin, rutin, myricetin, fisetin, quercitrin, quercetol, luteolin, kaempferol, apigenin, resveratrol, gallic acid, beta-resorcylic and epicatechin in several herbal extracts. Polyphenols were separated on a reverse phase Zorbax Eclipse Plus C18 column (3.0 × 100 mm × 3.5 µm). The LC-MS method enabled the screening and quantification of all extracts for 20 polyphenols, described by Ghiulai et al. 2020 [20]. The mobile phase consisted of a mixture of 0.1% acetic acid and methanol, used in gradient elution as follows: initially 5% methanol for the first 5 min (linear elution), gradually increasing to 42% methanol by minute 38, maintained at 42% methanol until minute 41, and returning to 5% methanol until minute 42.

The flow rate was set at 1 mL/min, achieving complete elution of all components within approximately 40 min. An injection volume of 10 µL was used, and the column temperature was maintained at 40 °C. Compound screening was performed using both UV and MS detectors. UV detection was conducted at wavelengths of 330 nm and 370 nm, while MS detection utilized electrospray ionization (ESI) in single ion monitoring mode (SIM) simultaneously for all 20 targeted compounds. All mass spectra were recorded in negative ion mode, previously established as the optimal ionization mode for these compounds [21]. Throughout the experiments, the capillary voltage was maintained at 3500 V, the drying gas flow rate was set at 12 L/min with a temperature of 350 °C, nebulizer pressure was maintained at 55 psig, and the fragmentor voltage was set at 70 V. For the quantitative determination of polyphenols in samples, calibration curves exhibiting good linearity were prepared using the external standard method over a concentration range of 0.05–2 µg/mL, with six calibration points for each compound. The mass spectrometer was calibrated externally using the ESI Tuning Mix standard from Agilent (Santa Clara, CA, USA).

### 2.3. Materials an Methods Used in the Formulations

All excipients and chemical reagents (sunflower oil, olive oil, isopropyl myristate, diethylene glycol monoethyl ether, glyceryl dibehenate—Compritol 888 ATO, Poloxamer 407, and glycerol) were obtained from the same commercial sources previously described by Rosca et al. (2025). Blank and plant extract-loaded oleogels and hydrogels were prepared exactly as previously described, using the specific proportions of ingredients explicitly presented here in Table 1.

### 2.4. Determination of Partition Coefficient LogP and Skin Permeability Potential

The evaluation of LogP and skin permeability potential was performed by high-performance liquid chromatography (HPLC). The experiments were conducted on a 6120 LC-MS analytical system from Agilent (Santa Clara, CA, USA) consisting of 1260 Infinity HPLC equipped with G1322A degasser, G1311B quaternary pump, G1316A column thermostat, G1365C MWD detector, G7129A autosampler coupled with a Quadrupolar (Q) mass spectrometer equipped with electrospray ionization source (ESI). OpenLAB CDS ChemStation Workstation software version C.01.08 was used to control the instrument, acquisition and processing the data. This was followed by in silico analysis using the SwissADME platform [22].

### 2.5. Physicochemical and Rheological Characterizationation Methods

The formulations were evaluated macroscopically (appearance, color, odor, opacity), by measuring pH (Seven Excellence™ S400-KIT, Mettler Toledo) and by analyzing rheological behavior (viscosity, thixotropy) using the HAAKE RheoStress 1 rheometer (Thermo Fisher Scientific). All experimental preparations were stored overnight at 4 °C and protected from light, before being submitted to physicochemical tests.

### 2.6. Methodology for Physicochemical and Rheological Characterization

#### 2.6.1. Determination of Macroscopic Properties and pH

Macroscopic evaluation of the experimental preparations was performed by visual examination of their organoleptic properties (physical appearance, color and homogeneity) with additional standardization of color using the Pantone Matching System [23,24], aspect [25,26] and detailed odor description based on sensory evaluation methods described by Lawless et al. [27,28,29,30].

The consistency of the gel formulations was evaluated by two related parameters: penetration degree and spreadability. The penetration degree was measured using a penetrometer (PNR 12, Petrolab, Speyer, Germany) equipped with a microcone, under standardized conditions according to the European Pharmacopoeia 11th edition method (Council of Europe, 2022). The spreadability was assessed using a del Pozo Ojeda-Suñé Arbussá extensometer following the parallel-plate method as previously described by Parente et al. (2015) [31,32]. All rheological measurements were performed at 25 ± 2 °C, in triplicate.

#### 2.6.2. Rheological Measurements

To determine the flow behavior and viscosity of experimental gels, a HAAKE RheoStress 1 rheometer (Thermo Fisher Scientific, Karlsruhe, Germany) with temperature device controller (TCP/P Peltier/Plate) was used. Viscosimetry tests were carried out at rotational mode and 23 °C, on a cone-plate geometry of 35 mm diameter, 2° cone angle and a 0.105 mm gap size for oleogels, and a plate–plate geometry of 60 mm diameter and a 1.000 mm gap size for hydrogels. The rheograms and viscosity curves were recorded under controlled rate by gradually increasing the share rate over a range of 0.05–100 1/s during 120 s (ramp-up period), 10 s at 100 1/s (constant shear rate period), and then down from the maximum to the minimum value of this range in another 120 s (ramp-down period). HAAKE RheoWin software version 4.3 (Thermo Fisher Scientific, Karlsruhe, Germany) was used to collect and analyze the rheological data.

#### 2.6.3. pH Analysis: Formulations and Burn Wounds

The pH values of experimental formulations and of a 10% DEGEE aqueous solution was measured in triplicate at 25 °C, using a calibrated Seven Excellence™ S400-KIT pH-meter (Mettler Toledo, Columbus, OH, USA) and according to the compendial potentiometric procedure [33]. From each gel, 1 g was dispersed in 20 mL purified water in a glass vial by stirring for 15 min, then the obtained dispersion was filtered, and the pH of the filtrate was determined. Results were expressed as mean ± SD.

### 2.7. Animal Study and Clinical Parameters

#### 2.7.1. In Vivo Study and Experimental Protocol

The in vivo experimental study was conducted on 30 female Wistar rats (250–300 g) under standard conditions, as previously described by Rosca et al. (2025) [34]. All experimental procedures strictly adhered to the ARRIVE guidelines [35] and were approved by the Ethics Committee of Victor Babes University of Medicine and Pharmacy, Timisoara (approval no. 17/11.03.2022).

#### 2.7.2. Anesthesia, Preoperative Preparation, and Pain Management

All procedures regarding animal anesthesia (ketamine/xylazine and isoflurane), monitoring, preoperative preparation (hair removal, depilatory cream, povidone-iodine solution), postoperative pain management (butorphanol administration), post-anesthetic recovery (temperature monitoring, crystalloid administration using the Parkland formula), and euthanasia were carried out exactly as previously described (Rosca et al., 2025) [34].

#### 2.7.3. Burn Induction and Wound Standardization

Burn wounds (second-degree scalds, 4% TBSA) were uniformly induced using the validated RAPID-3D device, applying boiling water (98–99 °C for 8 s) to defined skin areas, exactly as previously described and validated by Rosca et al. (2025) [34].

#### 2.7.4. Treatment Application and Dressing Protocol

Topical treatments (oleogels, hydrogels, and controls) and dressing protocols were applied exactly as previously described by Rosca et al. (2025) [34]. Briefly, each burn site was treated daily with 0.2 g of assigned formulations (Table 1), using sterile technique and a semi-occlusive dressing changed daily. Formulations were refrigerated at 4 °C throughout the study duration. Each experimental subgroup included untreated burn and healthy skin areas as controls, exactly as described previously.

#### 2.7.5. Statistical Analysis

Descriptive statistics (mean, standard deviation, median, range) were calculated for pH and burn surface area measurements. Pearson correlation coefficients were calculated to assess linear relationships between continuous variables, specifically average treatment pH (Days 1–21) or initial skin pH (Day 0) versus burn surface area at Day 21. To compare Day 21 burn surface area between burns categorized by average treatment pH (Acidic < 6 vs. 1 Basic > 7), the non-parametric Mann–Whitney U test was employed. For day-by-day analysis, wound pH was categorized (Acidic < 6, Neutral 6.0–7.0, Basic > 7.0) at each timepoint (Days 1, 4, 9, 14, 21) and burn surface area on the corresponding day was compared across these three categories using the non-parametric Kruskal–Wallis test. A *p*-value less than 0.05 was considered statistically significant for all tests.

## 3. Results

### 3.1. Quantitative Phytochemical Profile of Extracts

Phytochemical quantitative analysis measured the concentration of bioactive compounds in plant extracts: rutin (46.41 µg/g in ET-OH 99.5% of *Sambucus nigra* flowers), chlorogenic acid (18.68 µg/g in Sambucus nigra bark), rosmarinic acid (20.37 µg/g in ET-OH 99.5% of *Ocimum basilicum* and 3.47 µg/g in ET-OH 99.5% of *Galium verum*), and isoquercitrin (7.99 µg/g in *Sambucus nigra* flowers). *Boswellia serrata* (frankincense) extracts were characterized by notable amounts of quercitrin, epicatechin, and p-coumaric acid, known for their significant anti-inflammatory properties. Other identified phenolics include hyperoside, caffeic acid, and quercitrin, contributing further to the overall bioactive effects. The calculated LogP partition coefficient in silico indicates optimal permeability of most compounds through the skin, favorable for topical application.

**Figure 1:**
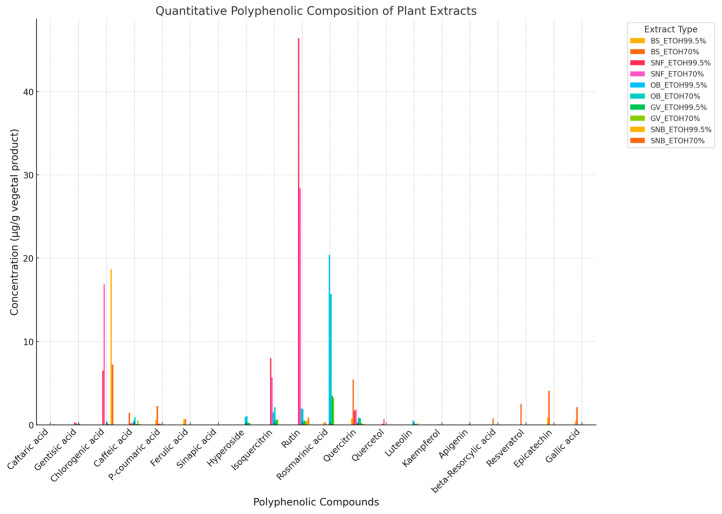
Comparative quantitative analysis of major phenolic compounds in selected plant extracts.

In Figure 1 is presented the quantitative analysis of phenolic compounds identified by HPLC in ethanolic (ET-OH 99.5) and hydroalcoholic (ET-OH 70%) extracts obtained from *Sambucus nigra flowers (SN)*, *Ocimum basilicum (OB)*, *Galium verum (GV)*, and *Sambucus nigra branch (SNB)*. Data is expressed in µg/g vegetal product and plotted on a logarithmic scale to emphasize differences across compounds and extracts. Major bioactive compounds such as rutin, chlorogenic acid, rosmarinic acid, and isoquercitrin exhibit the highest concentrations.

The calculated LogP values of the analyzed compounds were interpreted based on standard reference points, where values ranging from approximately −0.5 to 5 typically indicate favorable lipophilicity for efficient permeation through biological membranes (Lipinski et al., 2001) [36]. The permeability potential for skin penetration (Table 2) was assessed using the “500 Dalton rule,” stating that compounds with molecular weights below 500 Da generally exhibit higher skin permeability, whereas those above this threshold typically show lower permeability potential (Bos & Meinardi, 2000) [37].

### 3.2. Physicochemical and Rheological Properties

#### 3.2.1. Determination of Macroscopic Properties and pH

In Table 3, Table 4 and Table 5, the macroscopic properties (appearance, color, odor and transparency/opacity) of the experimental gel formulations are presented.

**Figure 2:**
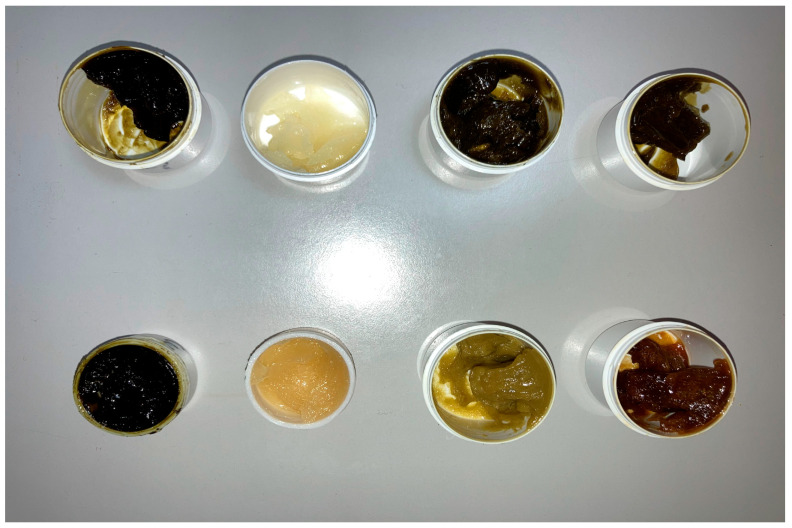
Presentation of the characteristics of hydrogel color, transparency, and opacity.

In Figure 2 is presented a photographic image that illustrates the hydrogels. For each sample can be observed the color, opacity or transparency.

#### 3.2.2. Standardized Macroscopic Evaluation of Topical Formulations

**Figure 3:**
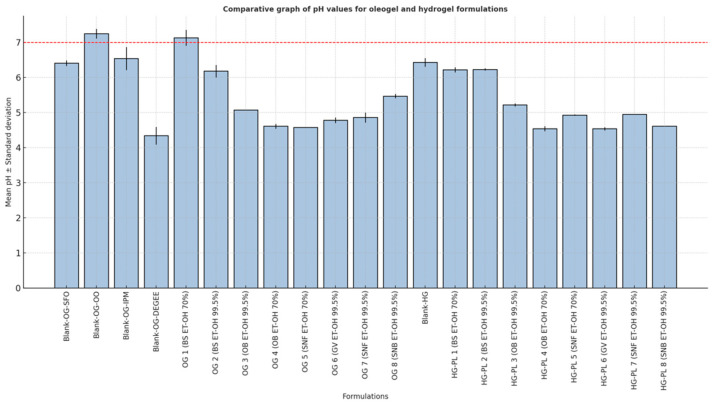
Determination of pH values for oleogel (OG) and hydrogel (HG) formulations.

Figure 3 presents a comparative graph of mean pH values (±standard deviation) for the experimental oleogel and hydrogel formulations. The values indicate that most of the formulations containing natural extract presented a more acidic pH compared to the corresponding topical control formulations, with the exception of OG1 (*Boswellia serrata* 70% ethanol), which presented neutral to slightly basic values. Diethylene glycol monoethyl ether (DEGEE)-based formulation recorded the lowest pH value (moderately acidic).

The dashed red line is the neutral pH value (pH = 7), thus facilitating a simple classification of the formulations as acidic, neutral or basic. All tested formulations presented pH values within the dermatologically acceptable range (4.5–8), suggesting adequate skin compatibility and a low risk of irritation.

### 3.3. Physicochemical Characterization

In the oleogels series, control formulations based on glyceryl dibehenate and various oily phases (sunflower oil, olive oil, isopropyl myristate, and diethylene glycol monoethyl ether—DEGEE) presented as soft, oily, white to yellowish-white, slightly to opaque, homogenous soft gels, with an odor characteristic of the liquid component of the composition.

When absolute ethanol or 70% ethanol plant extract was incorporated into the oleogel vehicle, the vegetal extract imprinted its characteristic color and smell. Similarly, in the poloxamer 407-based hydrogels series, the plant extract imprinted its specific color and smell to the control hydrogel, which was clear, colorless, and odorless. The measured pH values for all studied gels are depicted in Figure 3. Among the blank oleogel formulations, the systems containing sunflower oil, olive oil, and isopropyl myristate showed pH values between 6.41 ± 0.078 and 7.25 ± 0.136, indicating slightly acidic (Blank OG SFO and Blank OG IPM) and neutral (Blank OG OO) preparations. The pH of the oleogel based on DEGEE was lower (4.34 ± 0.251), indicating moderate acidity. This low pH value is attributed to DEGEE, a polar protic solvent. The pH of a 10% DEGEE aqueous solution measured within this study was 4.39 ± 0.028.

In sunflower oil-based oleogels, formulations OG2, OG3, and OG8 containing frankincense extract, basil extract, or elderberry bark extract in absolute ethanol showed moderately to slightly acidic pH values lower than the control oleogel, while formulation OG1 with frankincense extract in 70% ethanol presented a neutral pH slightly higher than the plain oleogel (Figure 3). Likewise, based on olive oil (OG6, OG7), IPM (OG4), and DEGEE (OG5) oleogels loaded with extracts exhibited lower pH values (between 4.58 ± 0.005 and 4.86 ± 0.144) compared to corresponding plain oleogels (Figure 3). For poloxamer 407-based hydrogels, formulations containing hydroalchoholic extract of frankincense (HG-PL 1) or absolute ethanol extract of frankincense (HG-PL 2) exhibited insignificantly different pH values (6.22 ± 0.075 and 6.23 ± 0.033) compared to the blank hydrogel (6.43±0.125). In contrast, HG-PL 3 and HG-PL 4 containing basil extract in absolute ethanol or 70% ethanol showed significantly lower pH values (5.22 ± 0.043 and 4.54 ± 0.073, respectively). Also, a lower pH values compared to the control hydrogel were observed for formulations HG-PL 5, HG-PL 7, and HG-PL 8 containing elderflower extract in 70% ethanol or absolute ethanol, and elderberry bark extract in absolute ethanol (4.93 ± 0.018, 4.95 ± 0.004, and 4.61 ± 0.005, respectively). Absolute ethanol extract of lady’s bedstraw also significantly decreased the hydrogel pH from 6.43 ± 0.125 to 4.54 ± 0.047 (Figure 3).

### 3.4. Rheological Characterization

**Figure 4:**
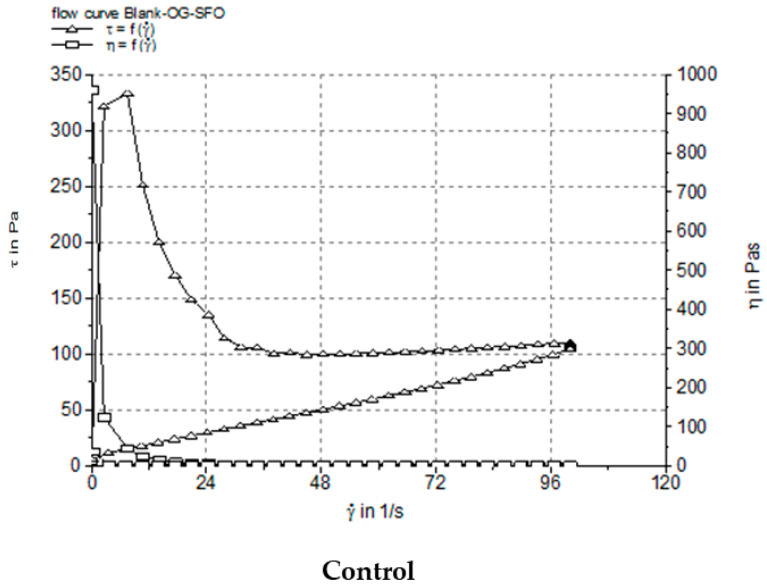
Rheological curves (tension and viscosity) of Blank-OG-SFO (control).

**Figure 5:**
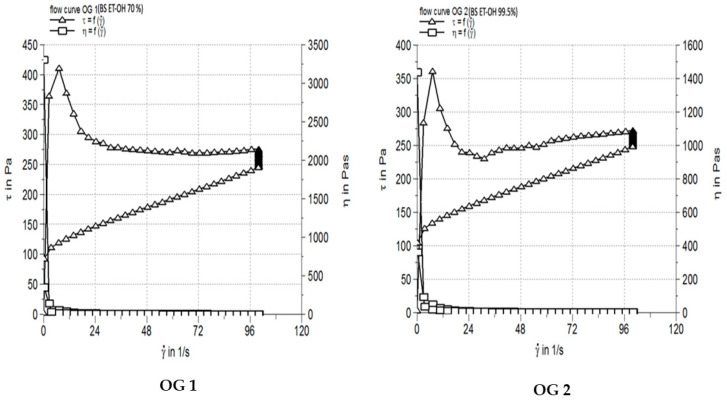
Comparative rheological curves (tension and viscosity) of formulations OG 1 (70% ethanol) and OG 2 (99.5% ethanol) based on frankincense extract and sunflower oil.

**Figure 6:**
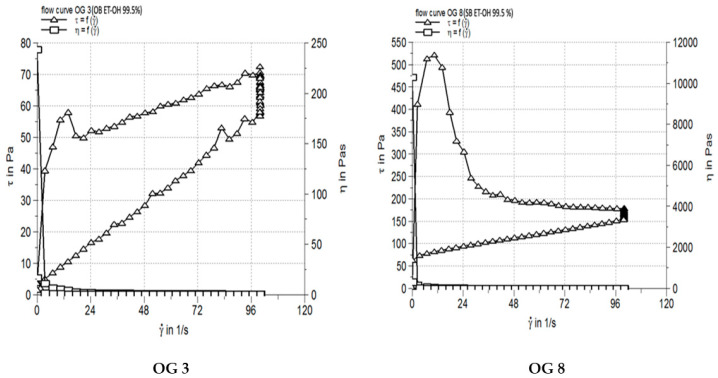
Comparative rheograms (shear stress and dynamic viscosity) of OG 3 (basil extract, 99.5% ethanol) and OG 8 (elder bark-branch extract, 99.5% ethanol) formulations versus Blank-OG-SFO.

**Figure 7:**
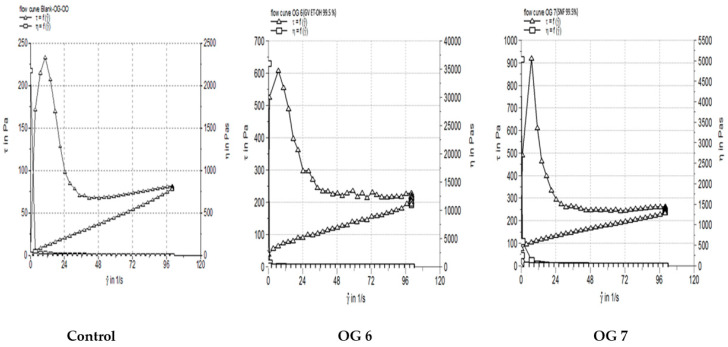
Comparative analysis of the rheological profile for olive oil-based oleogels loaded with plant extract, compared to the control formulation (Blank-OG-OO).

**Figure 8:**
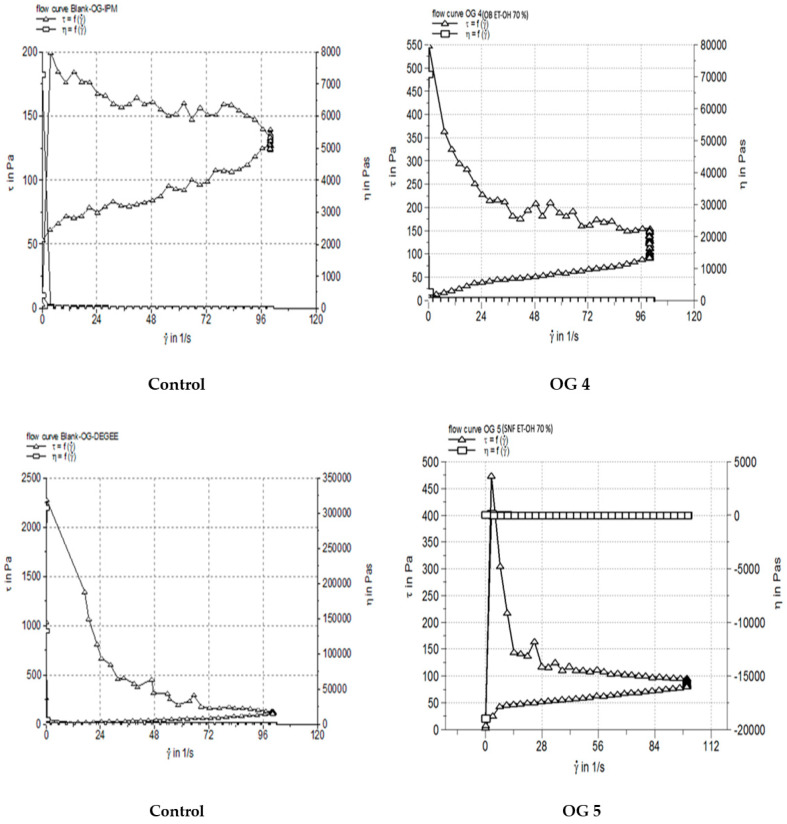
Flow and viscosity profiles of plant extract-loaded oleogels based on diethylene glycol monoethyl ether.

Rheological measurements (Figure 4, Figure 5, Figure 6, Figure 7 and Figure 8, Table 6) demonstrated that all plant extract-loaded gels exhibited pseudoplastic (shear-thinning) behavior, characterized by rapid viscosity reduction upon increasing shear rate.

The plant extract-loaded formulations had significantly higher initial viscosity and thixotropy than control gels, indicating enhanced applicability, improved persistence, and controlled bioactive compound release. Detailed rheological profiles, including viscosity and thixotropy data, are clearly presented in Figure 4, Figure 5, Figure 6, Figure 7 and Figure 8 and Table 6.

Rheological characterization of the sunflower oil-based oleogel (Blank-OG-SFO) as a control formulation for topical application.

The figure illustrates the brand a curve for the sample as “Blank-OG-SFO,” and the graph shows the importance of shear stress (τ, expressed in Pa) and dynamic viscosity (η, expressed in Pas) in terms of shear rate (γ, expressed in 1/s). Thus, in Figure 1, a rapid decrease in viscosity is observed with increasing shear rate, indicating a pseudoplastic rheological behavior (shear-thinning). In parallel, the shear stress gradually increases with increasing shear rate. These characteristics suggest that the analyzed sample is a viscoelastic formulation suitable for topical or cosmetic applications that require good application and surface area.

The formulations OG 1 and OG 2 show considerably higher initial values of shear stress (τ in Pa) and dynamic viscosity (η in Pas) compared to the control formulation (Blank-OG-SFO). The two samples, the formulation OG 1 (frankincense extract in 70% ethanol) shows the highest initial values, suggesting a higher viscosity and initial tension, which gives it a firmer consistency and a more adherent and persistent application on the skin surface compared to OG 2.

Both formulations demonstrate a clear pseudoplastic rheological behavior, characterized by a sharp decrease in viscosity as the shear rate increases (“shear-thinning”). The pseudoplastic behavior is more pronounced in the case of the OG 1 sample, suggesting that it has a more pronounced internal structure and a greater capacity to recover and maintain its shape after application, being suitable for applications requiring high skin protection, prolonged persistence, and a possible slow and sustained release of the active compounds.

In contrast, the OG 2 formulation (incense extract in 99.5% ethanol), although it has a high initial viscosity compared to the control (Blank-OG-SFO), is lower than those of the OG 1 sample. This makes OG 2 easier to distribute and has a lower persistence on the skin surface than OG 1, being more suitable for applications where faster absorption and easier distribution of the active substances are required.

Compared to the control formulation (Blank-OG-SFO), the two current samples (OG 3 and OG 8) show opposite rheological behaviors, with significant differences in the initial values of shear stress (τ) and dynamic viscosity (η).

The formulation OG 3 (basil extract in 99.5% ethanol) shows much lower initial values of stress and viscosity than the control Blank-OG-SFO, indicating a more fluid consistency, with much easier application and rapid distribution on the skin surface. This formulation is ideal for applications where rapid absorption and a light feeling on application are preferred.

In contrast, the formulation OG 8 (elder bark extract in 99.5% ethanol) demonstrates the highest initial tension and viscosity values among all the previously analyzed formulations (Blank-OG-SFO, OG 1, OG 2 and OG 3). The extremely high initial viscosity of OG 8 suggests an intensely adherent formulation capable of ensuring long-term persistence and controlled release of active compounds, which is ideal for therapeutic applications requiring prolonged and protective effects on the skin.

Compared to the control formulation (Blank-OG-OO), the formulations OG 6 and OG 7 demonstrate higher initial values of shear stress (τ in Pa) and dynamic viscosity (η in Pas). In particular, the formulation OG 6 (Gallium verum extract in ethanol 99.5%) presents the highest initial values, indicating a very viscous and intensely adherent consistency, which suggests its potential to ensure long-term persistence and a controlled and slow release of active substances at the skin level.

The formulation OG 7 (elderflower extract in ethanol 99.5%) has lower initial viscosity and tension values than OG 6 but higher than the control (Blank-OG-OO). The intermediate rheological profile makes it suitable for applications that require a balance between adhesion, persistence, and ease of distribution on the skin.

Comparing the control formulations, those based on isopropyl myristate and DEGEE were more viscous than those based on vegetable oils (sunflower oil or olive oil), whereas their degree of thixotropy varied into a relatively narrow domain (5845–8019 Pa/s), except the DEGEE-based oleogel presenting a considerably higher thixotropy (54,860 Pa/s) (Table 2). In the group of sunflower oil-based oleogels, the formulations with frankincense extract (OG 1 and OG 2) presented similar viscosity values, being two times more viscous than the plain formulation, but differed in thixotropy, since the thixotropic character of OG 1 was slightly more pronounced than those of OG 2 and of plain oleogel (Table 2). Also, the oleogel loaded with absolute ethanol extract of elderberry bark (OG 8) showed a 1.5-fold higher viscosity and 1.7-fold higher thixotropy than control formulation. Instead, the oleogel containing basil extract in absolute ethanol produced the lowest viscosity and thixotropy values in this group of oleogels (Table 2). The oleogels loaded with lady’s bedstraw or elderflower extract in absolute ethanol (OG 6, respectively, OG 7 formulations) were 2.5 to 3 times more viscous and thixotropic than the corresponding plain oleogel. However, when 70% ethanolic extract of basil was loaded in IPM-based oleogel, a slight decrease in viscosity and 2.3 times increase in thixotropy were observed. Similarly, the 70% ethanolic extract of elderflower decreased slightly the viscosity of DEGEE-based oleogel but decreased drastically its thixotropy (7 folds) (Table 2).

On the other hand, the viscosity values of all studied poloxamer-based hydrogels were approximately an order of magnitude higher than those of the oleogels (Table 2). In the group of hydrogels, the formulations containing 70% ethanolic extracts of basil and, respectively, elderflower (HG-PL 4 and HG-PL 5) showed the lowest viscosity, followed closely by the formulations with absolute ethanol extracts of elderberry bark and lady’s bedstraw (HG-PL 8 and HG-PL 6). However, when extracts of frankincense, basil or elderflower in absolute ethanol were loaded into the poloxamer-based hydrogel, systems similar in viscosity with control hydrogel were obtained. The hydrogel containing frankincense extract in 70% ethanol was the most viscous among all experimental hydrophilic gels.

Additionally, the rheograms of these gels presented hysteresis loops, revealing their thixotropic behavior. Incorporation of ethanolic extracts in poloxamer-based hydrogel significantly increased the thixotropy value, except for the extract of elderberry bark, for which no influence on this parameter was observed. Compared to blank hydrogel, there was a 1.5–3-fold increase in thixotropy values of HG-PL 6, HG-PL 7, HG-PL 3 and HG-PL 4 hydrogel formulations, while in case of HG-PL 5, HG-PL 2 and HG-PL 1 formulations the increase was greater (7.8–9.5 times).

**Figure 9:**
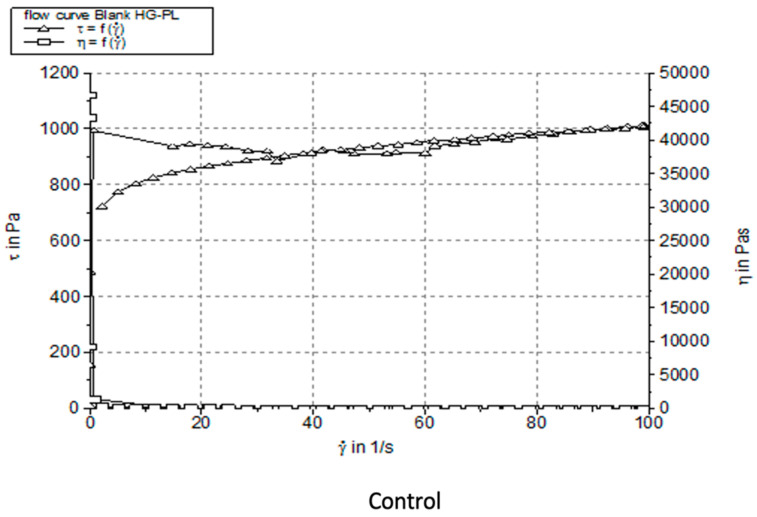
Analysis of the rheological profile of the Poloxamer 407-based control hydrogel (Blank HG-PL) intended for topical applications.

According to Figure 9, the above (Blank HG-PL) shows the rheological profile of a Poloxamer 407-based control hydrogel. The formulation demonstrates extremely high initial values for shear stress (τ in Pa) and dynamic viscosity (η in Pas), indicating a very pronounced viscous consistency. The observed rheological behavior is typically pseudoplastic (“shear thinning”), reflected by a slight decrease in viscosity with increasing shear rate, which can provide significant persistence in the skin and is suitable for topical applications requiring stability over time, high adhesion and a slow and controlled release of active substances.

**Figure 10:**
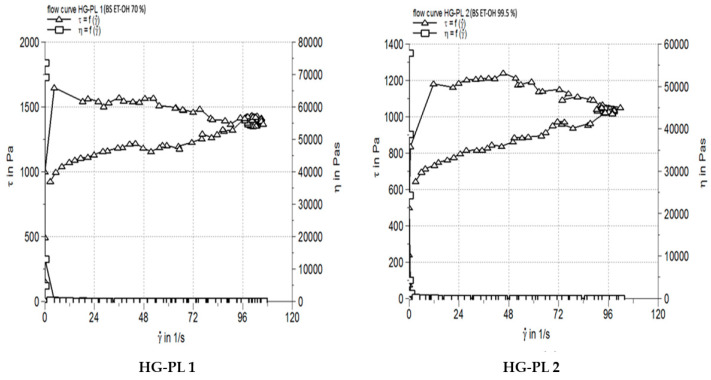
Comparative rheological profiles (shear stress and dynamic viscosity) of the hydrogels HG-PL 1 (incense extract in 70% ethanol) and HG-PL 2 (incense extract in 99.5% ethanol) compared to the control hydrogel (Blank HG-PL).

Figure 10 proves that the first two hydrogels analyzed (HG-PL 1 and HG-PL 2) present higher initial values for both shear stress (τ in Pa) and dynamic viscosity (η in Pas) compared to the Blank HG-PL.

In particular, the hydrogel HG-PL 1 (frankincense extract in 70% ethanol) demonstrates the highest initial viscosity and shear stress values, indicating a highly viscous, strongly adherent, and stable formulation. This makes the hydrogel HG-PL 1 ideal for applications requiring high persistence, stability, and controlled, slow release of active compounds on the skin surface.

Although the hydrogel HG-PL 2 (incense extract in 99.5% ethanol) shows lower viscosity and initial tension values than HG-PL 1, it is still more viscous than the control (Blank HG-PL). Thus, this formulation can ensure good adhesion and applicability and is suitable for topical applications that balance adhesion and easy distribution.

**Figure 11:**
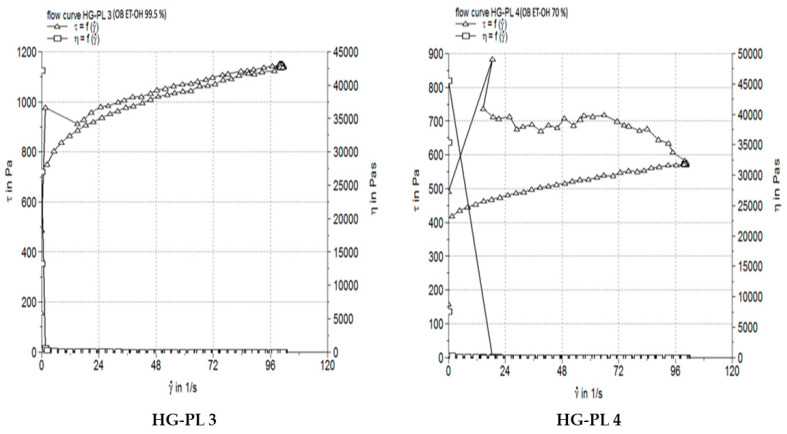
Comparative flow and viscosity profiles of the hydrogels HG-PL 3 (basil extract in 99.5% ethanol) and HG-PL 4 (basil extract in 70% ethanol) compared to the control hydrogel (Blank HG-PL).

The formulations HG-PL 3 and HG-PL 4 (see Figure 11) show relatively close initial values, but are still slightly lower in terms of shear stress (τ in Pa) and dynamic viscosity (η in Pas). Compared to the control hydrogel (Blank HG-PL). The formulation HG-PL 3 (basil extract in 99.5% ethanol) presents initial values of viscosity and stress close to the control hydrogel, suggesting that this formulation maintains a high consistency, suitable for applications requiring good adhesion and persistence on the skin.

In contrast, the formulation HG-PL 4 (basil extract in 70% ethanol) shows noticeably lower initial values, demonstrating a lower viscosity and more fluid behavior. This characteristic suggests that HG-PL 4 would be more suitable for topical applications where rapid distribution and easy absorption are important.

**Figure 12:**
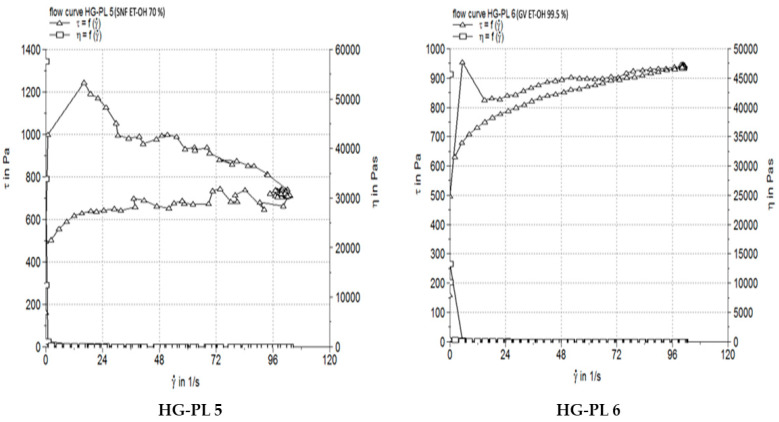
Comparative analysis of the rheological profiles for Poloxamer 407-based hydrogels loaded with 70% ethanol extracts of Lady’s bedstraw (HG-PL 5) and 99.5% ethanol extracts of elderflower (HG-PL 6), compared to the control hydrogel.

The Lady’s bedstraw 70% ethanol hydrogel extract (HG-PL 5) demonstrates higher initial values of shear stress (τ in Pa) and dynamic viscosity (η in Pas) (see Figure 12), indicating a significantly more viscous formulation, with improved adhesion and stability compared to the control formulation (Blank HG-PL). These properties make the HG-PL 5 hydrogel suitable for applications requiring long-term persistence and slow and controlled release of active principles.

In contrast, the hydrogel HG-PL 6 (elderflower extract, ethanol 99.5%) has lower initial viscosity and tension values, close to the control’s, suggesting a more fluid consistency and easier applicability (see Figure 12). This formulation is ideal for topical applications where faster absorption and comfortable distribution on the skin are preferred.

**Figure 13:**
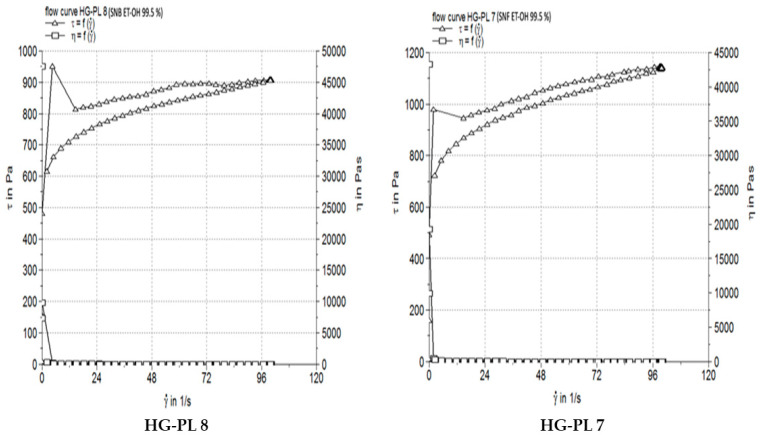
Comparative analysis of rheological profiles for Poloxamer 407-based hydrogels loaded with elder bark-branch extracts in 99.5% ethanol (HG-PL 8) and elder flowers in 99.5% ethanol (HG-PL 7), compared to the control hydrogel (Blank HG-PL).

Compared to the control hydrogel (Blank HG-PL), the current formulations (HG-PL 7 and HG-PL 8) present initial values of shear stress (τ in Pa) and dynamic viscosity (η in Pas) relatively similar to the control, indicating close consistencies and comparable rheological behaviors (see Figure 13).

The HG-PL 7 hydrogel (elder flower extract, 99.5% ethanol) presents initial viscosity values and stress similar to or slightly lower than the control hydrogel. This suggests a stable formulation, suitable for topical applications that require comfortable applicability and easy distribution on the skin.

The HG-PL 8 hydrogel (elder bark-branch extract, ethanol 99.5%) demonstrates slightly higher initial viscosity and tension values than the control, indicating a slightly more adherent and stable consistency, which could support prolonged persistence and slow release of the active compounds.

The consistency or the structural strength of a semisolid material is the resistance exhibited by a sample to deformation by an applied force. Consistency has two main components, which can be quantified, namely the hardness and the spreadability, both being important properties for topical formulations. The hardness is usually measured by penetrometry and expressed as penetration depth (in mm), based on the inversely proportional relationship between them (a harder semisolid exhibits a lower penetration depth). The spreadability of a topical formulation is usually measured by parallel plate method and is considered an important characteristic, because of its ease of application on the skin and extrudability from the package.

The penetration depth values exhibited by the four plain oleogels were slightly higher than the upper limit generally recommended for white petrolatum (300 mm), which is extensively used as excipient and ointment base for pharmaceutical semisolids. On the other hand, the incorporation of ethanolic vegetal extracts in the oleogel vehicles determined in all cases an approximately 3-fold decrease in the penetration values, which indicates the increase in the consistency of the formulations; however, the formulation with basil extract in absolute ethanol (OG 3) was only 1.4 times more consistent than the oleogel base (penetration value 256.3 ± 4.73 mm versus 370.0 ± 7.24) (Table 6). Similarly, all the plant extract-loaded poloxamer based hydrogels produced lower penetration values than that of plain hydrogel, revealing their higher consistency (Table 6). The differences between these formulations can be attributed primarily to the different chemical composition of the plant extracts and, secondarily, to their ethanolic concentration.

**Figure 14:**
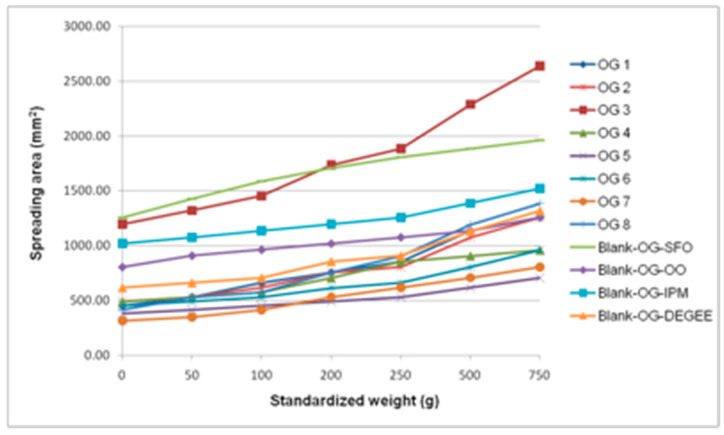
Spreadability profiles of plant extract-loaded oleogels.

**Figure 15:**
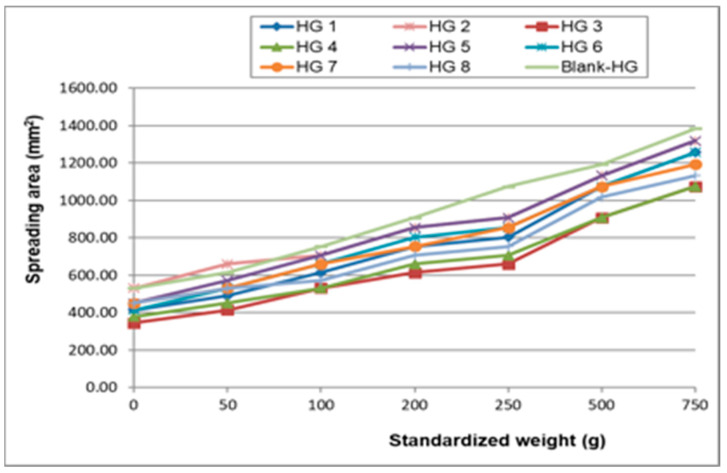
Spreadability profiles of plant extract-loaded poloxamer-based hydrogels.

The results of the spreadability test are presented (Figure 14 and Figure 15) by plotting the spreading area (in mm^2^) of gels as function of applied weight (in g) at 25 °C.

Under the pressure of a 750 g weight, the blank oleogel based on SFO showed the highest spreading area (1962.50 ± 2.35 mm^2^) and consequently the best spreadability among all oleogel bases, whereas the oleogel bases containing IPM, DEGEE or OO produced lower spreading areas (1519.76 ± 1.47 mm^2^, 1319.59 ± 2.28 mm^2^ and, respectively, 1256.00 ± 3.11 mm^2^) (Figure 14). Except for the OG 3 formulation, significant lower spreadability values were measured for all extract-loaded oleogel formulations with respect to those obtained for the corresponding plain oleogels, indicating greater consistency and viscosity (Figure 14). In contrast, the OG 3 formulation presented a higher area of spreadability than that of plain oleogel (2640.74 ± 3.51 mm^2^ versus 1962.50 ± 2.35 mm^2^), being softer and less viscous (Figure 14).

The effect of decreasing vehicle’s spreadability in the presence of the plant extract was also observed in the series of poloxamer-based hydrogels. Thereby, the values of the spreading area measured for all plant extract-loaded hydrogels varied into a relatively narrow range (from 1074.67 ± 2.85 mm^2^ to 1319.59 ± 1.44 mm^2^) but were lower than that of the plain hydrogel (1384.74 ± 1.69 mm^2^).

Overall, despite the observed differences between plain and extract-loaded gels, it is worth mentioning that all these systems were characterized by appropriate consistency (hardness) and spreadability.

### 3.5. Comparative pH Evaluation

#### 3.5.1. Association Between Wound pH Category and Burn Surface Area at Specific Timepoints

To investigate the potential influence of the wound pH environment on healing progression at discrete timepoints, the measured pH at each burn site was categorized as Acidic (<6), Neutral (6.0–7.0), or Basic (>7.0) on Days 1, 4, 9, 14, and 21 post-burn. Burn surface area (mm^2^) was compared across these pH categories for each respective day using the Kruskal–Wallis test.

On Day 1, all burn surface areas were uniformly 200 mm^2^, precluding statistical comparison across pH categories. On Day 4, no significant difference in burn surface area was observed among the pH categories (*p* = 0.297).

**Figure 16:**
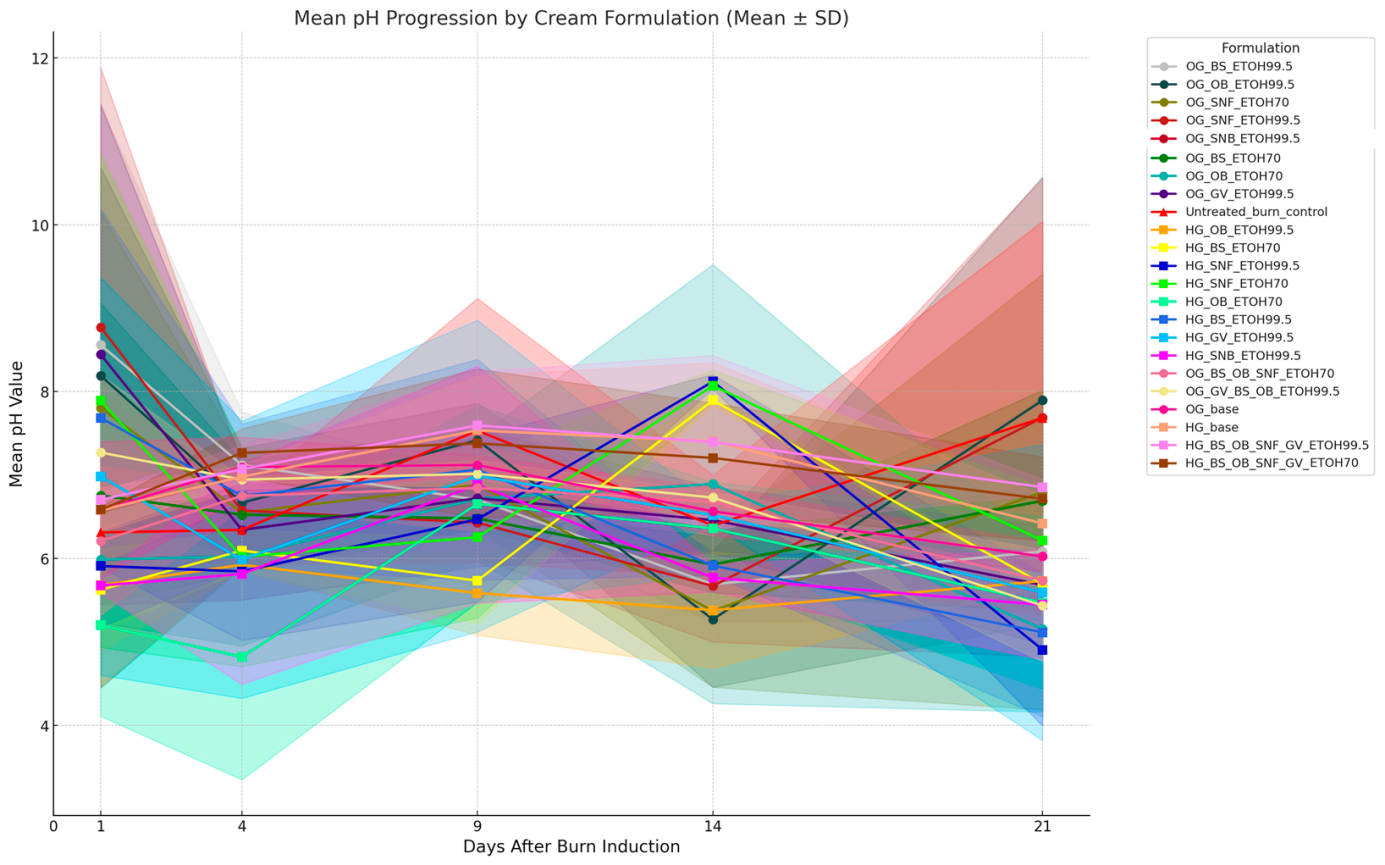
Progression of skin surface pH over time by topical cream formulation.

Figure 16 illustrates the progression of mean skin surface pH values (±standard deviation) over a 21-day period following burn induction for topical cream formulations containing different plant extracts (*Ocimum basilicum*—OB, *Sambucus nigra*—SN, *Boswellia serrata*—BS, *Gallium verum*—GV), formulated as oleogels (OG) and hydrogels (HG) with varying ethanol (EtOH) concentrations.

**Figure 17:**
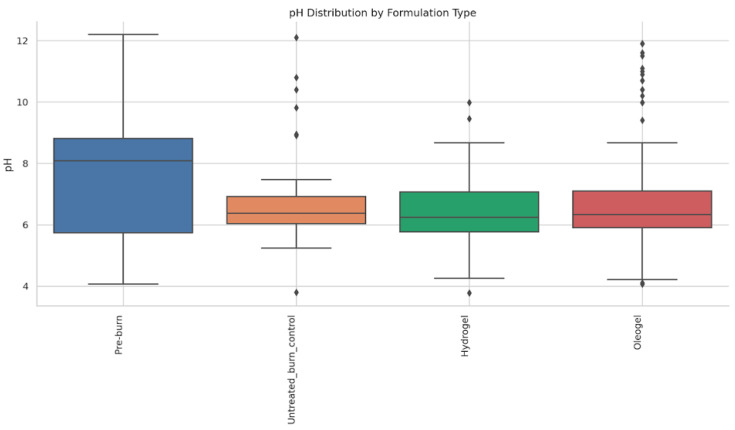
Skin surface pH distribution by treatment formulation type before and after burn injury.

Box plot illustrating the distribution of skin surface pH values grouped by treatment formulation types: pre-burn (baseline), untreated burn control, hydrogel, and oleogel formulations (Figure 17). Horizontal lines within boxes represent median values; boxes indicate interquartile ranges; whiskers show the range excluding outliers, and diamonds represent outliers. The variability in pH among different formulations indicates the potential impact of treatment type on the skin’s pH and consequently on the healing environment.

**Figure 18:**
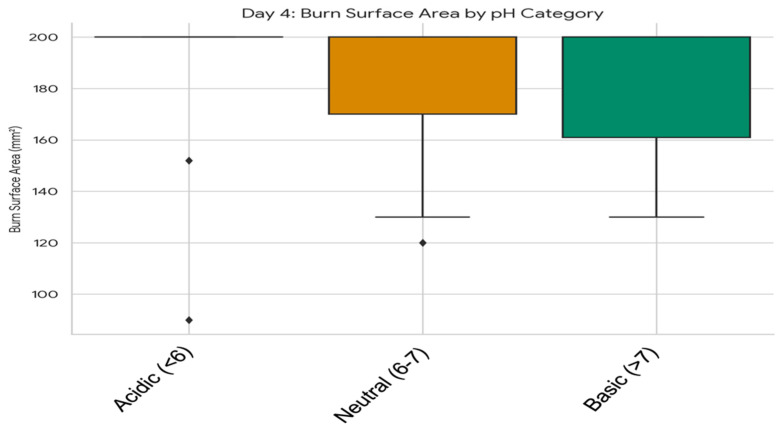
Effect of topical formulation pH on burn surface area at Day 4 post-burn.

Box plot illustrating the burn surface areas (mm^2^) at day 4 post-injury, categorized by topical formulation pH: acidic (pH < 6), neutral (pH 6–7), and basic (pH > 7). Boxes represent the interquartile range, horizontal lines within boxes denote median values, whiskers show the range of the data excluding outliers, and diamonds indicate outliers. Lower surface area values indicate better healing progression (see Figure 18). 

**Figure 19:**
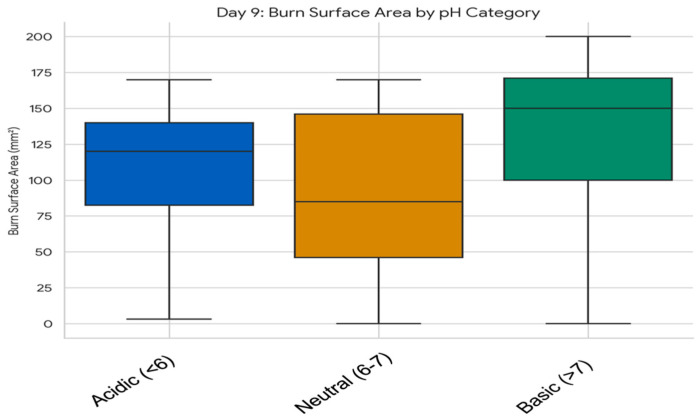
Effect of topical formulation pH on burn surface area at Day 9 post-burn.

**Figure 20:**
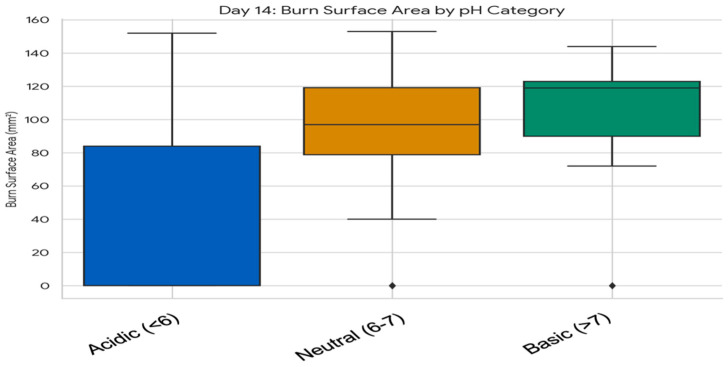
Effect of topical formulation pH on burn surface area at Day 14 post-burn.

However, statistically significant differences emerged at intermediate timepoints. On Day 9, a significant difference in burn surface area was found across pH categories (*p* = 0.025). Visual inspection of the data distribution (See Figure 19) suggested that burns exhibiting an acidic pH (<6) on this day generally had smaller surface areas compared to those with neutral or basic pH. This association was more pronounced on Day 14, where a highly significant difference in burn surface area was detected among pH categories (*p* < 0.001). Burns measured with an acidic pH (<6) on Day 14 consistently showed smaller surface areas relative to burns with neutral or basic pH (See Figure 20).

Box plot showing burn surface areas (mm^2^) measured on day 14 post-injury, categorized by the pH of applied topical formulations: acidic (pH < 6), neutral (pH 6–7), and basic (pH > 7). The horizontal lines inside the boxes represent median values; boxes indicate interquartile ranges; whiskers show the range of data excluding outliers, while diamonds indicate outliers. Smaller surface area values correspond to improved burn healing.

By Day 21, although considerable healing had occurred across all groups, the differences in burn surface area among the pH categories measured on this final day were no longer statistically significant (*p* = 0.673).

These findings indicate a statistically significant association between an acidic wound environment (pH < 6) and reduced burn surface area during the intermediate stages (Days 9 and 14) of the observation period in this model.

**Figure 21:**
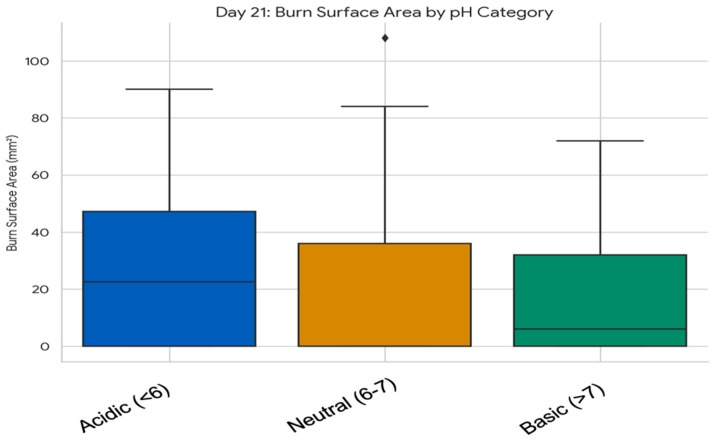
Effect of topical formulation pH on burn surface area at Day 21 post-burn.

Box plot illustrating burn surface areas (mm^2^) at day 21 post-injury, grouped by pH category of topical formulations: acidic (pH < 6), neutral (pH 6–7), and basic (pH > 7). The horizontal lines within each box indicate median burn surface areas, boxes represent the interquartile range, whiskers represent the range excluding outliers, and the diamond shape indicates an outlier. Lower values indicate better healing outcomes with smaller burn surface areas (see Figure 21).

**Figure 22:**
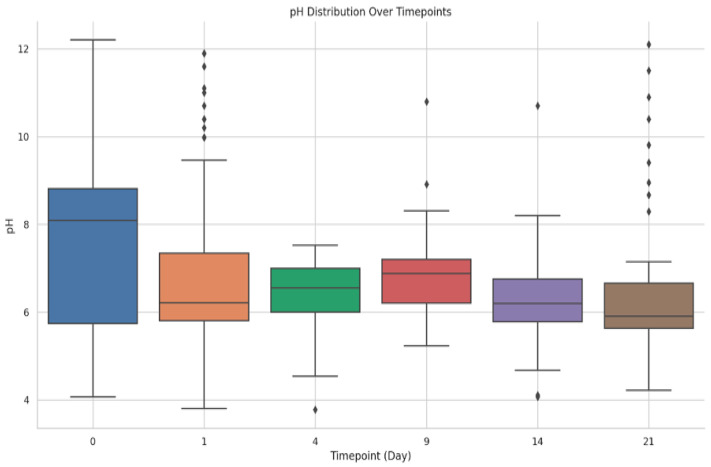
Evolution of pH values in formulations across different timepoints.

The boxplot represents the distribution and stability of pH values measured at multiple timepoints (Day 0, 1, 4, 9, 14, and 21) throughout the experimental period (see Figure 22). Boxes indicate the interquartile range (25th–75th percentiles), the central line represents the median, whiskers denote the variability outside the upper and lower quartiles, and points beyond whiskers represent outliers. The observed stabilization of pH values indicates formulation stability over time.

**Scheme 1:**
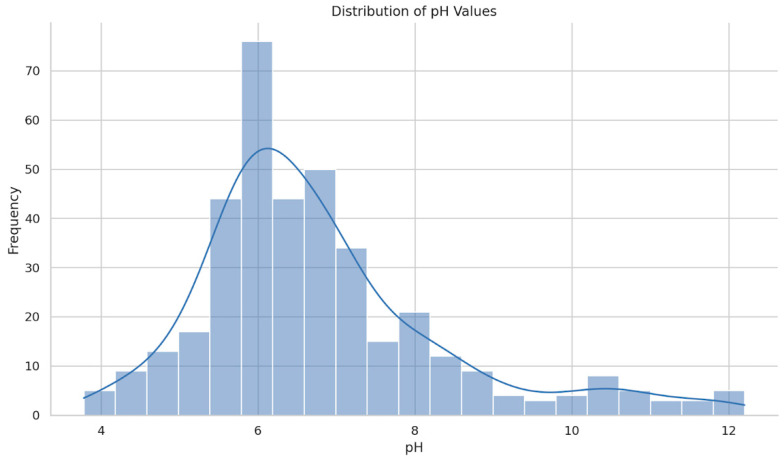
Distribution of skin surface pH values measured following the application of various topical formulations during the post-burn period. The histogram is complemented by a density curve illustrating the general distribution trend of the data.

Scheme 1 illustrates the frequency distribution of skin surface pH measurements after the application of studied topical formulations. Most pH values are concentrated between 5 and 7, showing a clear peak around neutral to slightly acidic (~pH 6). Extreme values, either highly acidic (below pH 4) or highly basic (above pH 10), are infrequent. This distribution suggests that most topical formulations help maintain a relatively neutral or mildly acidic skin environment, favorable for post-burn skin healing.

#### 3.5.2. Comparative Analysis of Skin Surface pH and Temperature Dynamics in Relation to Plant Extracts and Formulation Types

The *Sambucus nigra* bark extract in absolute ethanol (HG_SNB_99.5%) is distinguished by the most stable and consistently acidic pH value, suggesting the best compatibility with the physiological requirements of skin healing compared to another *Sambucus nigra* formulation extract. On the other hand, topical formulation with *Ocimum basilicum* (HG_OB_70%), its constant acidic stability, recommending it as the most suitable for therapeutic application in wound treatment, due to its superior compatibility with the physiological environment. The pH trend in *Ocimum basilicum* formulations appears to be dependent on the extraction solvent (see Figure 16).

*Galium verum* in absolute ethanol (HG_GV_99.5% and OG_GV_99.5%) demonstrates efficient adaptation and final stabilization in an acidic environment, indicating good therapeutic potential (see Figure 16).

All another formulations extract, regardless of solvent and formulation, show significant variations, suggesting the need for further optimization for clinical use.

The burn surface area decreases clearly and consistently over time, demonstrating effective wound healing.

**Figure 23:**
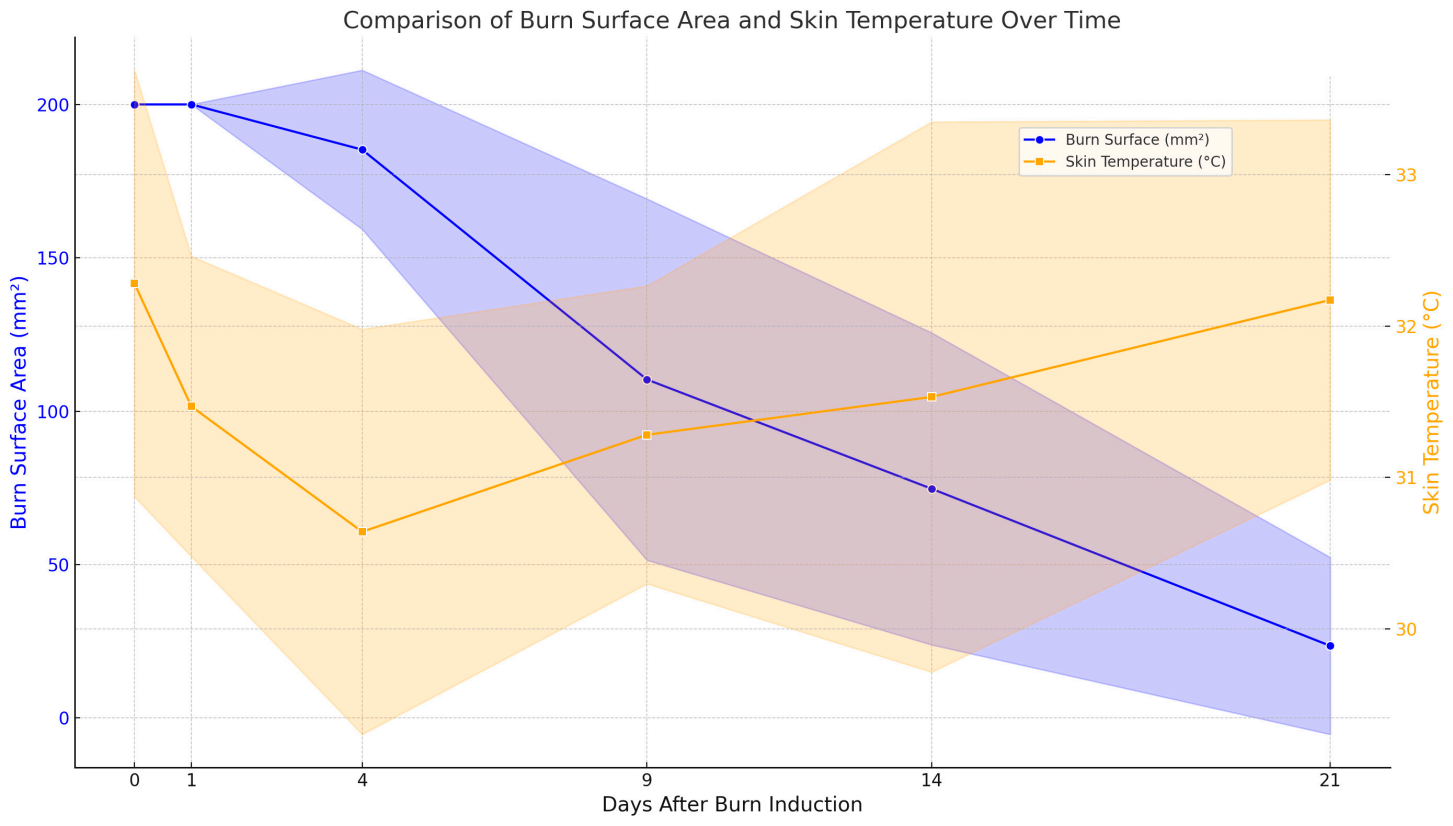
Comparison of wound surface evolution and skin temperature over time (mean ± SD).

Skin temperature shows some fluctuation but does not display a strong or clear decreasing/increasing trend directly corresponding to the healing progression. Instead, temperature appears relatively stable, with minor fluctuations (Figure 23).

**Figure 24:**
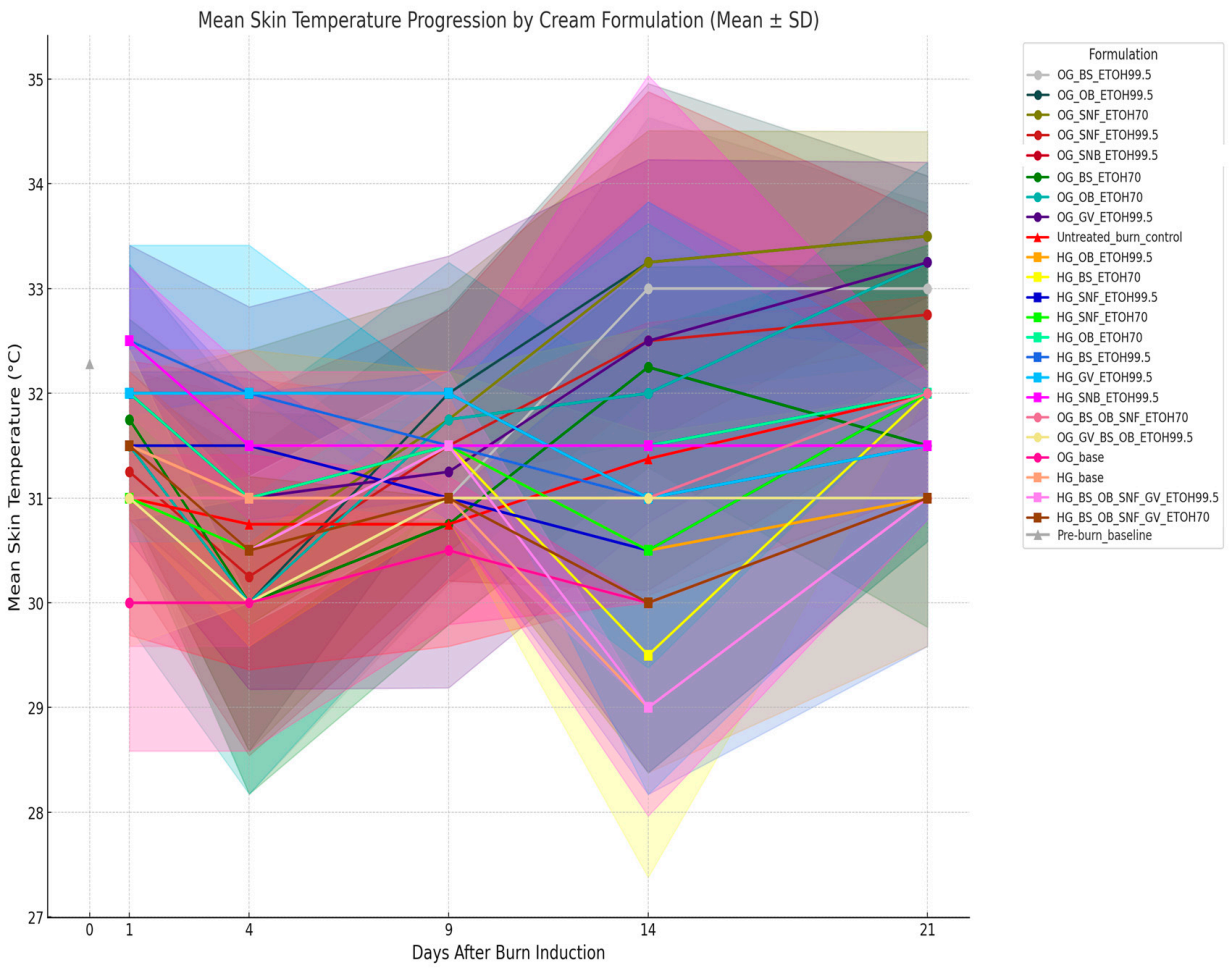
Evolution of mean skin temperature after burn induction, depending on the topical formulation used (mean ± SD).

The dynamics of the mean skin temperature (in °C), measured on days 0, 1, 4, 9, 14, and 21 after experimental induction of a scald burn using various topical formulations (oleogels—OG and hydrogels—HG). The basal temperature before the burn (“Pre-burn baseline”) is used as a reference (Figure 24).

Initially (day 0), the mean skin temperature in the burned area is increased compared to the basal level, reflecting the inflammation and hyperemia associated with the thermal injury. First days after the burn induction (day 1 to day 4), most formulations lead to a slight decrease in local temperature, which could suggest a reduction in the local inflammatory process.

Between days 4 and 9, the temperature evolution differs between the oleogel and hydrogel formulations. Formulation without incorporated extracts maintaining a constant temperature around 30–31 °C compared with others that show more pronounced variations (e.g., OG_OB_ETOH70, HG_OB_ETOH99.5).

A sign that the inflammatory process is attenuated and wound healing is approaching complete epithelialization are shown on day 14 and 21 where most formulations converge towards a temperature close to the basal value. However, formulations HG_OB_ETOH70 and HG_OB_ETOH99.5 seem to maintain a slightly higher temperature, which may indicate a prolonged inflammatory process compared to other formulations.

These data suggest that the type and composition of topical formulations directly influence the burn wound’s skin temperature. This temperature indirectly correlates with the degree of inflammation and, implicitly, with the evolution of the healing process. Formulations with a faster reduction and maintenance of values closer to baseline may indicate superior efficacy in controlling inflammation and accelerating healing.

### 3.6. Clinical and Histological Evaluation of Burn Healing

**Figure 25:**
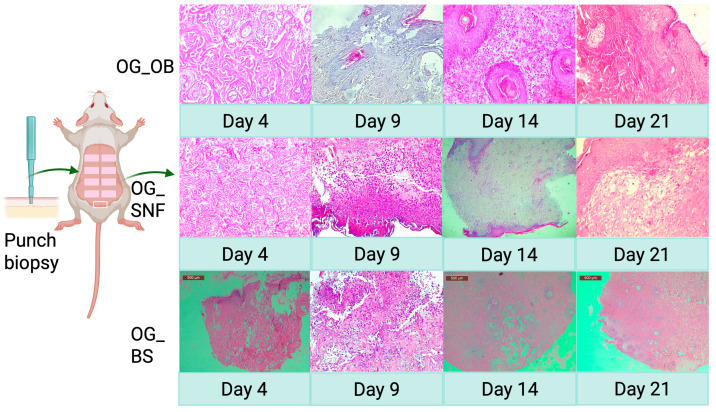
Histological evolution of the healing process of burn wounds in rats depending on the applied extract (OB—*Ocimum basilicum*, SNF—*Sambucus nigra* flos, BS—*Boswellia serrata*). Created in BioRender by Rosca Oana-Janina, 2025, https://BioRender.com/cdu78mo (accessed on 21 June 2025).

Oleogel applications accelerated the general healing process, as previously demonstrated by Rosca et al. [18]. Consistent with our earlier findings, individual formulations containing *Ocimum basilicum* and *Boswellia serrata* demonstrated the highest efficacy in reducing wound surface area, histologically enhancing epithelialization, and granulation while also reducing inflammation (see Figure 25). In this study, we further observed that formulations with more basic pH values (e.g., *Ocimum basilicum*-based oleogels) led to histologically increased fibrosis and deep inflammation, potentially indicating a higher risk of hypertrophic scarring.

Additionally, hydrogel formulations demonstrated clinical performance similar to that of the oleogels; notably, the hydrogel formulation containing *Sambucus nigra* flower extract exhibited explicit and significant effects on vascularization and epithelialization, which correlated directly with a progressive decrease in pH toward acidic values—an observation newly described here.

#### 3.6.1. Impact of pH Variations in Hydrogel Formulations on Vascularization, Epithelialization, and Inflammation

Regarding the hydrogel formulations, it is observed that SNF 99.5%, from an acidic pH at rheological characterization and day 0 tends towards neutral-basic values starting with day 4 to day 14, progressively decreasing to an acidic pH on day 21. From a histopathological evolutionary point of view, it presents many neoformation vessels and an advanced epithelialization process observed on day 14.

On the other hand, the BS 99.5% extract from a slightly acidic rheological pH to a basic-neutral pH from day 0 to day 9, and from day 14, it becomes acidic. Histopathologically, an accentuated epithelialization process is noted, with less fibrosis from day 14, but with the maintenance of a slight inflammatory syndrome also on day 21.

#### 3.6.2. Correlation Between Formulation pH, Rheological Characteristics, and Healing Progression

Statistical analysis revealed a significant correlation between acidic formulation pH and reduction in wound surface area on days 9 and 14 (*p* < 0.05). pH values accelerate the healing compared to neutral or basic formulations.

Topical products exhibiting higher initial viscosity and thixotropy showed enhanced persistence and stability on the wound surface, promoting sustained release of bioactive compounds, clinically translating into improved epithelialization, reduced inflammation, and overall faster healing processes.

## 4. Discussion

The study findings underscore the critical importance of maintaining a slightly acidic skin environment during post-burn wound healing. Our observations align with recent literature indicating that an acidic wound pH supports antimicrobial activity, maintains skin barrier integrity, and accelerates epithelialization and granulation processes [38,39].

To the best of our knowledge, this is the first study to explicitly integrate simultaneous analyses of phytochemicals, physicochemical parameters (pH, temperature), and detailed rheological parameters, offering a comprehensive perspective on burn wound healing.

An interesting observation in our study was that although the total concentration of plant extracts remained identical at 5%, formulations containing a combination of four plant extracts (1.25% each of *Boswellia serrata*, *Ocimum basilicum*, *Sambucus nigra*, and *Galium verum*) appeared to be less effective than those with single-plant extracts. This finding suggests a possible reduction in synergistic effects or even potential antagonistic interactions among bioactive compounds when these extracts are combined. Such results highlight the importance of careful selection and evaluation of combined herbal formulations, as therapeutic efficacy may not necessarily correlate directly with total extract concentration, but rather with specific phytochemical interactions.

The extracts plant used in the present study, particularly, *Boswellia serrata* and *Sambucus nigra*, demonstrated significant capacity to dynamically regulate the pH local, in direct correlation with the histological and clinical outcomes.

Due to their abundant boswellic acids, *Boswellia serrata* extracts, are reported in the literature to have an anti-inflammatory effect by inhibiting the 5-lipoxygenase pathway and reducing chronic inflammatory infiltrate.

Additionally, the presence of rutin, chlorogenic acid, and rosmarinic acid in *Sambucus nigra* and *Galium verum* significantly contributes to reducing local oxidative stress and stimulating collagen synthesis, essential aspects of tissue regeneration.

The rheological properties of the formulations, notably their high viscosity and thixotropy, facilitated a sustained and stable release of bioactive compounds, directly contributing to the clinically observed therapeutic efficacy. Previous studies confirm that topical formulations exhibiting pseudoplastic and thixotropic behavior ensure optimal adherence and prolonged persistence on wounds, thus enabling gradual and sustained release of active substances.

### 4.1. Role of LogP and Additional Physicochemical Parameters in Assessing Skin Permeability

The classification of LogP values in relation to skin permeability of compounds as hydrophilic, optimally permeable, or lipophilic can represent a determining factor in dermatopharmacological practice based on the understanding that the octanol–water partition coefficient can serve as an indicator of a compound’s ability to penetrate the stratum corneum [40].

Although the numerical thresholds are commonly used, Log *p* < 1 for hydrophilic, 1–3 for optimal permeability, and >3 for lipophilic, in the pharmacological branch, they are used as guides in developing topical formulations [41].

The study conducted by Chen et al. (2018) [41] showed a strong correlation between LogP values and skin permeability coefficients (Kp), demonstrating that compounds with higher LogP values tend to have increased skin permeability.

Although LogP is a significant factor in determining skin permeability, it is not the only determinant regarding physicochemical properties [42]. Also, in vitro clinical data and those on human subjects must be correlated, for example, with molecular weight, hydrogen bonding capacity, and molecular size, as well as complex analyses related to polarity, solubility, and experimental data using Franz cells or Raman confocal microscopy to understand better the mechanism of skin permeability of the evaluated topical formulations [43,44].

Integrating the phytochemical, physicochemical, rheological, and permeability characteristics studied herein provides a robust rationale for the observed clinical efficacy. Specifically, optimized LogP values enhance skin penetration of active components, aligning with the controlled release characteristics facilitated by the rheological properties of the formulations. Furthermore, the resulting pH modulation synergizes with these physicochemical aspects, collectively creating an optimal environment conducive to accelerated wound healing.

### 4.2. Correlation of Rheological Assessment in Literature Topical Formulation

The flow and viscosity curves depicted in Figure 1, Figure 2, Figure 3 and Figure 4 confirmed the non-Newtonian thixotropic pseudoplastic (shear-thinning) behavior of all analyzed gels. The thixotropy is displayed by the hysteresis-loop referring to the system capacity to recover its viscosity in time when the flow is discontinued applied, and the specific pseudoplastic behavior is indicated by a fall in viscosity with an increasing share rate [45]. All studied gels (oleogels and hydrogels) showed a time-dependent decrease in the viscosity determined by the shear rate, which is considered a typical rheological behavior for such systems [46,47]

This sudden drop in viscosity to relatively low shear rate values can be attributed to the shear sensitivity of the oleogels, which can be correlated with weak interactions forces within their tridimensional network. The pseudoplastic flow is a desirable characteristic for a pharmaceutical topical gel as it improves the preparation’s spreadability on the skin.

The values of viscosity and degree of thixotropy (Table 2) for all oleogel systems (control and extract-loaded formulations) show the influence of the composition and polarity of both oleogel liquid phase and plant extracts on the gel microstructure, as was previously reported [48].

Different extents of thixotropy displayed by the experimental extract-loaded hydrogels suggest changes in the level of interactions responsible for the gel structural network most probably caused by the components of the extracts, since it was previously evidenced that ethanol affects the poloxamer gel formation only in concentrations higher than 20% [49,50].

### 4.3. Clinical Significance of pH and Temperature Monitoring in Effective Wound Healing Management

Wound healing is a process that involves numerous biochemical and physiological parameters, among which pH and temperature are extremely important [51]. Investigation and monitoring of indicators can generate valuable information about lesions overall favorable or unfavorable status, with the potential for maintaining inflammation, the likelihood of bacterial colonization of the wound, and these two parameters [52].

Changing the pH of the wound can have a major impact on wound evolution. Healthy skin maintains the protective barrier at an optimal pH between 4.1 and 5–8. A deviation towards an alkaline pH (>6.5) can signal a bacterial infection or vicious healing conditions [53,54].

The importance of temperature variations in wound management is equally critical.

The increase in temperature of an acute injury is also due to local vascular activity, by providing oxygen and nutrients essential for restoring the integrity of the protective barrier [55,56].

These in healing wounds tend to be slightly higher than in normal skin, but sudden temperature increases indicate infection and an increased inflammatory response, while temperature decreases may indicate compromised circulation and ischemia [57]. Fluctuations of up to 2.2 °C can serve as warning signs of impending wound deterioration, underscoring the importance of continuous and accurate temperature monitoring [58,59].

Although temperature and pH monitoring in our study were on days 0, 4, 9, 14, 21 our results are also supported by the observations of Ning Tang et al. (2021) [52 showing that continuous, real-time monitoring of pH and temperature parameters using sensors in the wound dressing offers significant clinical and patient comfort advantages in the effective management of wound healing.

Furthermore, research by Graziela Heberlé et al. (2012) and Eneida Janiscki Da-Lozzo et al. (2011) indicates that topical formulations, such as emulgels containing bioactive ingredients such as curcuminoids or cranberry extracts, can further influence wound microenvironment parameters such as pH and temperature [60,61].

### 4.4. Comparative Analysis with Existing Plant-Based Hydrogels and Oleogels

Various plant-based hydrogels and oleogels have been extensively studied, focusing on their wound-healing effects due to their antioxidant, anti-inflammatory, and antimicrobial properties.

Gavan et al. (2022) developed carbomer-based hydrogels loaded with extracts of *Rosmarinus officinalis*, *Achillea millefolium*, and *Calendula officinalis*, demonstrating promising antimicrobial activities and suitable rheological properties for wound management. They highlighted rosemary’s role in enhancing granular tissue formation and collagen deposition. At the same time, *Achillea* and *Calendula* showed significant anti-inflammatory and antimicrobial effects [62].

Andleeb et al. (2021) formulated nanoethosomes loaded with *Achillea millefolium* extract into topical gels, revealing enhanced skin permeation and stability with significant antioxidant activities attributed to flavonoid and phenolic content [63]. Similarly, Serbezeanu et al. (2021) presented electrospun polyvinyl alcohol nanofibers incorporating plant extracts, demonstrating strong antioxidant and antimicrobial effects beneficial for wound healing applications [64].

Alexa et al. (2022) explored natural emulsions based on *Thymus vulgaris*, *Origanum sativum*, and *Coriandrum sativum*, which exhibited substantial antifungal properties and inhibited mycotoxin production. These emulsions were noted for their stability and effectiveness in preserving wheat grains, indirectly supporting their applicability in topical antimicrobial and protective formulations [65].

Parihar and Verma (2023) evaluated novel emulgels containing extracts of *Symphytum officinale* (Comfrey), *Achillea millefolium* (Yarrow), and *Commiphora myrrha* (Myrrh), demonstrating excellent wound closure rates and desirable physicochemical characteristics, which emphasize their holistic therapeutic potential due to their combined antimicrobial, anti-inflammatory, and regenerative effects [66].

Recent studies have shown the promising role of plant extracts in wound care. Turcov et al. (2022) elaborated on dermato-cosmetic formulations containing antioxidants from *Galium verum*, which combat oxidative stress in the skin and promote effective dermatological applications [67].

Antonescu et al. (2021) demonstrated the wound healing potential of hydrogels based on combined extracts of *Ocimum basilicum* and *Trifolium pratense*, showing enhanced fibroblast proliferation in vitro and improved wound closure in vivo [68].

In the same way, Khan et al. (2020) formulated an emulgel based on *Ocimum basilicum*, highlighting its significant efficacy in burn wound healing comparable to commercial products [69]. Furthermore, Mota et al. (2020) reported the anti-inflammatory properties of *Sambucus nigra* extracts encapsulated in PLGA and PCL nanoparticles, showcasing advanced therapeutic potential through nanoformulation [70].

In comparison, the present study introduces unique hydrogel and oleogel systems tailored specifically to modulate wound pH and temperature, crucial parameters influencing healing efficiency. By incorporating carefully selected extracts from *Boswellia serrata*, *Ocimum basilicum*, *Sambucus nigra*, *and Galium verum*, our formulation maintains an acidic environment beneficial for epidermal barrier repair, modulation of inflammation, and inhibition of microbial growth. The specific selection and combination of bioactive extracts, coupled with optimized physicochemical and rheological properties, provide a targeted and comprehensive approach to burn treatment, extending beyond the scope of previously studied formulations on wound healing.

### 4.5. Limitations and Future Directions

Our study demonstrates robust therapeutic outcomes; nevertheless, certain inherent aspects merit further exploration. An extended observation period might provide additional insights into long-term healing outcomes such as scar formation and tissue remodeling. Although the Wistar rat model is well-validated, additional human clinical trials are necessary to confirm direct clinical applicability.

The pH values obtained in this study could be influenced by certain specific endogenous and exogenous factors, potentially affecting the accuracy of results, as indicated by other studies [71,72]. Thus, further research is required to analyze the pH of topical creams considering all known factors from specialized literature on the studied plants. Additionally, a comparative analysis of adjusted versus unadjusted pH results could provide valuable dermatopharmacological guidance for developing topical formulations intended as potential treatments for burns or other inflammatory-based lesions in both human and veterinary medicine. Moreover, future studies should also focus on characterizing the diffusion of topical formulations into the skin concerning the molecular weight of the compounds present in plant extracts.

Additional limitations of this study include the limited duration of observation, which may not fully capture long-term healing outcomes such as final scarring or tissue remodeling.

The animal model used, although widely accepted, has physiological differences compared to human skin, which could limit the applicability of the results to clinical human settings.

Moreover, the lack of direct and detailed microbiological assessments in the study restricts understanding of specific antimicrobial activities of the tested formulations.

Additionally, the study did not explore varying concentrations of plant extracts, leaving unanswered questions regarding optimal therapeutic dosages. Lastly, interindividual variability (genetic, metabolic) influencing healing and treatment responses was not addressed.

Future research directions should include controlled clinical trials on human populations to validate efficacy and safety, extended monitoring to evaluate long-term scarring outcomes, detailed microbiological evaluations, comparative studies on different extract concentrations, and advanced molecular characterization to elucidate the underlying healing mechanisms. Moreover, studies aimed at understanding the molecular interactions between bioactive compounds within mixed plant extracts would clarify and potentially enhance the efficacy of formulations. Furthermore, studies examining individual variability and combination therapies, as well as economic feasibility analyses, would further strengthen the clinical application potential of these topical formulations.

## 5. Conclusions

The present study demonstrates the efficacy of oleogel and hydrogel formulations containing plant extracts (*Boswellia serrata*, *Sambucus nigra, Galium verum* and *Ocimum basilicum*) as a potential first-line treatment in experimental post-burn wound dressings that may facilitate wound healing. The main findings include

Phytochemical analysis revealed significant bioactive compounds (boswellic acids, rutin, chlorogenic acid, rosmarinic acid) that contribute to anti-inflammatory, antioxidant and regenerative effects.

Optimized physicochemical properties (appropriate pH and LogP values) significantly improved skin permeability and supported healing processes.

Rheological properties (high viscosity and thixotropy) ensured efficient application, prolonged persistence and sustained release of active substances.

Clinical and histological evaluations confirm accelerated healing, improved epithelialization, reduced inflammation and favored tissue regeneration, directly correlating with the properties of the formulation.

These results emphasize that the phytochemical composition, optimal physicochemical properties, and favorable rheological profiles make these formulations candidates for advanced clinical management.

The present study offers clear and evidence-based insights into the design and formulation of clinically efficacious topical formulations, highlighting their potential as tailored wound dressings to enhance therapeutic outcomes in burn care.

## Figures and Tables

**Table 1:** Detailed description of oleogel and hydrogel formulations containing ethanolic extracts.

Formulation	Extraction Method	Extract and Concentration (*w*/*w*)	Gel Components	In Vivo Unique Name	Description
OG	ETOH99.5	OB 5%	Sunflower oil 80% Glyceryl dibehenate 15%	OG_OB_ETOH99.5	Oleogel with *Ocimum basilicum* extract in absolute ethanol, based on glyceryl dibehenate and sunflower oil
OG	ETOH99.5	BS 5%	Sunflower oil 80% Glyceryl dibehenate 15%	OG_BS_ETOH99.5	Oleogel with *Boswellia serrata* extract in absolute ethanol, based on glyceryl dibehenate and sunflower oil
OG	ETOH99.5	SNF 5%	Olive oil 80% Glyceryl dibehenate 15%	OG_SNF_ETOH99.5	Oleogel with *Sambucus nigra* flower extract in absolute ethanol, based on glyceryl dibehenate and olive oil
OG	ETOH99.5	SNB 5%	Sunflower oil 80% Glyceryl dibehenate 15%	OG_SNB_ETOH99.5	Oleogel with *Sambucus nigra* bark brunch extract in absolute ethanol, based on glyceryl dibehenate and sunflower oil
OG	ETOH99.5	GV 5%	Olive oil 80% Glyceryl dibehenate 15%	OG_GV_ETOH99.5	Oleogel with *Galium verum* extract in absolute ethanol, based on glyceryl dibehenate and olive oil
OG	ETOH70	OB 5%	Isopropyl myristate 80% Glyceryl dibehenate 15%	OG_OB_ETOH70	Oleogel with *Ocimum basilicum* extract in 70% ethanol, based on glyceryl dibehenate and isopropyl myristate
OG	ETOH70	BS 5%	Sunflower oil 80% Glyceryl dibehenate 15%	OG_BS_ETOH70	Oleogel with *Boswellia serrata* extract in 70% ethanol, based on glyceryl dibehenate and sunflower oil
OG	ETOH70	SNF 5%	Diethylene glycol monoethyl ether 80% Glyceryl dibehenate 15%	OG_SNF_ETOH70	Oleogel with *Sambucus nigra* flower extract in 70% ethanol, based on glyceryl dibehenate and diethylene glycol monoethyl ether
OG	ETOH99.5	BS_OB_SNF_GV 1.25:1.25:1.25:1.25%	Sunflower oil 20% Olive oil 20% Isopropyl myristate 20% Diethylene glycol monoethyl ether 20% Glyceryl dibehenate 15%	OG_BS_OB_SNF_GV_ETOH99.5	Oleogel with 4 plant extracts in absolute ethanol
OG	ETOH70	BS_OB_SNF_GV 1.25:1.25:1.25:1.25%	Sunflower oil 20% Olive oil 20% Isopropyl myristate 20% Diethylene glycol monoethyl ether 20% Glyceryl dibehenate 15%	OG_BS_OB_SNF_GV_ETOH70	Oleogel with 4 plant extracts in 70% ethanol
HG	ETOH99.5	OB 5%	Purified water 60% Poloxamer 407 25% Glycerol 10%	HG_OB_ETOH99.5	Hydrogel with *Ocimum basilicum* extract in absolute ethanol
HG	ETOH99.5	BS 5%	Purified water 60% Poloxamer 407 25% Glycerol 10%	HG_BS_ETOH99.5	Hydrogel with *Boswellia serrata* extract in absolute ethanol
HG	ETOH99.5	SNF 5%	Purified water 60% Poloxamer 407 25% Glycerol 10%	HG_SNF_ETOH99.5	Hydrogel with *Sambucus nigra* flower extract in absolute ethanol
HG	ETOH99.5	GV 5%	Purified water 60% Poloxamer 407 25% Glycerol 10%	HG_GV_ETOH99.5	Hydrogel with *Galium verum* extract in absolute ethanol
HG	ETOH70	OB 5%	Purified water 60% Poloxamer 407 25% Glycerol 10%	HG_OB_ETOH70	Hydrogel with *Ocimum basilicum* extract in 70% ethanol
HG	ETOH70	BS 5%	Purified water 60% Poloxamer 407 25% Glycerol 10%	HG_BS_ETOH70	Hydrogel with *Boswellia serrata* extract in 70% ethanol
HG	ETOH70	SNF 5%	Purified water 60% Poloxamer 407 25% Glycerol 10%	HG_SNF_ETOH70	Hydrogel with *Sambucus nigra* flower extract in 70% ethanol
HG	ETOH99.5	SNB 5%	Purified water 60% Poloxamer 407 25% Glycerol 10%	HG_SNB_ETOH99.5	Hydrogel with *Sambucus nigra* bark brunch extract in absolute ethanol
HG	ETOH70	BS_OB_SNF_GV 1.25:1.25:1.25:1.25%	Purified water 60% Poloxamer 407 25% Glycerol 10%	HG_BS_OB_SNF_GV_ETOH70	Hydrogel with 4 plant extracts in 70% ethanol
HG	ETOH99.5	BS_OB_SNF_GV 1.25:1.25:1.25:1.25%	Purified water 60% Poloxamer 407 25% Glycerol 10%	HG_BS_OB_SNF_GV_ETOH99.5	Hydrogel with 4 plant extracts in absolute ethanol
Pre-burn	N/A	Baseline	N/A	Pre-burn_baseline	Control for the healthy skin
Untreated_ Burn	N/A	Untreated_Burn	N/A	Untreated_burn_control	Control for the untreated burn
OG	N/A	OG_base	Sunflower oil 21.5% Olive oil 21.5% Isopropyl myristate 21.5% Diethylene glycol monoethyl ether 21.5% Glyceryl dibehenate 14%	OG_base	Control oleogel (without bioactive component)
HG	N/A	HG_base	Purified water 65% Poloxamer 407 25% Glycerol 10%	HG_base	Control hydrogel (without bioactive component), based on poloxamer 407 and glycerol, purified water

**Table 2:** Molecular weight, permeability potential, and Log Po/w values of selected bioactive compounds.

No.	Compound Name	Molecular Weight (g/mol)	Permeability Potential	Log Po/w
1	Caftaric acid	311	High	−0.29
2	Gentisic acid	153	High	0.74
3	Chlorogenic acid	353	High	−0.43
4	Caffeic acid	179	High	0.93
5	P-coumaric acid	163	High	1.26
6	Ferulic acid	193	High	1.36
7	Sinapic acid	223	High	1.31
8	Hyperoside	463	High	−0.38
9	Isoquercitrin	463	High	−0.48
10	Rutin	609	Low	−1.51
11	Rosmarinic acid	359	High	5.15
12	Quercitrin	447	High	−0.05
13	Quercetol	301	High	1.23
14	Luteolin	285	High	1.73
15	Kaempferol	285	High	1.58
16	Apigenin	269	High	2.11
17	Gaelic acid	169	High	0.21
18	Epicatechin	289	High	0.83
19	Beta-Resorcylic	153	High	0.77
20	Resveratrol	227	High	2.48

**Table 3:** Standardization of formulation color using pantone codes methods.

Qualitative Description (Original)	Suggested Pantone Reference
Pearly white	Pantone 11-0601
Straw yellow	Pantone 13-0922
White	Pantone 11-0601
Dull-yellow	Pantone 13-0850
Orange	Pantone 16-1364
Green-moss	Pantone 17-0530
Brown	Pantone 18-0930
Pale brown	Pantone 15-1213
Brownish-dark	Pantone 19-1015
Brownish-green	Pantone 18-0435
Colorless	Pantone Transparent

**Table 4:** Standardized scale for opacity/transparency.

Term Used	Definition	Example Formulations
Transparent	Completely clear, no turbidity	Blank-HG
Translucent	Slightly cloudy but still clear	HG-PL 3–8
Slightly opaque	Mild turbidity, partially opaque	Blank-OG-SFO, HG-PL 1, HG-PL 2
Opaque	Completely opaque, no transparency	OG 1–8, Blank-OG-OO, Blank-OG-IPM, Blank-OG-DEGEE

**Table 5:** Suggested standardization of aromatic descriptions.

Original Odor Description	Improved Standardized Description
Sunflower oil characteristic	Mild vegetal, characteristic of sunflower oil
Olive oil characteristic	Mild vegetal, characteristic of olive oil
Isopropyl myristate characteristic	Neutral, characteristic of IPM
DEGEE characteristic	Slightly chemical, neutral odor
Specific, slightly aromatic	Mild herbal aroma
Specific aromatic	Herbal, fresh aroma
Specific aromatic (brownish)	Earthy, herbal aroma
Specific aromatic (brown-dark)	Woody, earthy aroma
Odorless	Odorless (neutral)

**Table 6:** Viscosity, thixotropy and penetration depth measured for the studied plant extract-loaded oleogels and hydrogels.

Formulation	Viscosiy (Pa·s)	Thixotropy (Pa/s)	Penetration Depth (mm)
Blank-OG-SFO	1.071733 ± 0.014	8019	370.0 ± 7.24
Blank-OG-OO	0.799833 ± 0.009	5845	321.0 ± 8.07
Blank-OG-IPM	1.3042 ± 0.041	7367	339.0 ± 3.65
Blank-OG-DEGEE	1.217 ± 0.081	54,860	330.0 ± 6.41
OG 1	2.583 ± 0.084	10,850	107.0 ± 5.64
OG 2	2.586 ± 0.066	6977	106.7 ± 5.03
OG 3	0.640 ± 0.041	2900	256.3 ± 4.73
OG 4	1.185 ± 0.182	17,370	113.0 ± 2.28
OG 5	0.867 ± 0.043	7717	103.7 ± 1.53
OG 6	2.062 ± 0.091	17,300	118.0 ± 2.14
OG 7	2.457 ± 0.081	15,910	113.7 ± 1.53
OG 8	1.645 ± 0.068	13,710	130.0 ± 2.66
Blank HG-PL	10.179 ± 0.027	9932	126.3 ± 1.58
HG-PL 1	13.855 ± 0.415	94,340	84.7 ± 3.05
HG-PL 2	10.839 ± 0.275	88,420	85.3 ± 2.52
HG-PL 3	11.389 ± 0.097	17,410	97.7 ± 1.15
HG-PL 4	5.723 ± 0.028	30,040	99.3 ± 1.53
HG-PL 5	7.274 ± 0.187	77,680	119.7 ± 0.58
HG-PL 6	9.341 ± 0.055	14,740	113.3 ± 2.08
HG-PL 7	11.467 ± 0.059	15,360	112.0 ± 1.05
HG-PL 8	9.073 ± 0.019	10,470	117.3 ± 2.52

## Data Availability

The original contributions presented in this study are included in the article. Further inquiries can be directed to the corresponding author(s).

## Abbreviations

The following abbreviations are used in this manuscript:

Blank-OG-OO	Blank oleogel olive oil formulation
BS	*Boswellia serrata*
Compritol 888 ATO	Glyceryl dibehenate excipient
DEGEE	Diethylene glycol monoethyl ether
GV	*Galium verum*
HG	Hydrogel
LC-MS	Liquid chromatography-mass spectrometry
LogP	Logarithm of partition coefficient
OC	*Ocimum basilicum*
OG	Oleogel
pH	Potential of hydrogen
PVP-I	Liposome polyvinyl-pyrrolidone-iodine
RAPID-3D	Rat printed induction device-3D
SD	Standard deviation
SNB	*Sambucus nigra* brunch bark
SNF	*Sambucus nigra* flower
SFO	Sunflower oil
TA	Topical antimicrobial
